# One-Dimensional Carbon Nanostructures with Periodic Graphitic Nitrogen Substitution

**DOI:** 10.1021/acsnano.6c12638

**Published:** 2026-10-30

**Authors:** Nicolò Bassi, Shantanu Mishra, Zheng Zhang, Xiao-Ye Wang, Feifei Xiang, Nils Krane, Carlo A. Pignedoli, Klaus Müllen, Pascal Ruffieux, Akimitsu Narita, Roman Fasel

**Affiliations:** † nanotech@surfaces Laboratory, 542594 Empa—Swiss Federal Laboratories for Materials Science and Technology, 8600 Dübendorf, Switzerland; ‡ Department of Physics and Astronomy, 11248 Chalmers University of Technology, 412 96 Göteborg, Sweden; § Organic and Carbon Nanomaterials Unit, Okinawa Institute of Science and Technology Graduate University, 904-0495 Okinawa, Japan; ∥ 12538 Max Planck Institute for Polymer Research, 55128 Mainz, Germany; ⊥ College of Chemistry, 28501 Nankai University, 300071 Tianjin, P. R. China; # Department of Chemistry, Biochemistry and Pharmaceutical Sciences, University of Bern, 3012 Bern, Switzerland

**Keywords:** scanning tunneling microscopy, atomic force microscopy, on-surface synthesis, density functional theory, heteroatoms, substitutional doping

## Abstract

Heteroatom substitution is a powerful route to tune the chemical and electronic properties of carbon nanomaterials. In particular, replacement of an sp^2^-hybridized carbon atom in the graphene lattice with a nitrogen atom (denoted as graphitic nitrogen) induces substantial changes in the electronic properties. These include changes in the band structure that can influence electronic transport and magnetism. A key requirement for applications is both the periodic and precise incorporation of the heteroatoms in extended carbon lattices. Here, we report the on-surface synthesis and characterization of two one-dimensional carbon nanostructures—a polymer and a graphene nanoribbon—consisting of periodically incorporated graphitic nitrogen atoms. The on-surface reactions toward the formation of the nanostructures were monitored by scanning tunneling microscopy. The bond-resolved chemical structures of the reaction intermediates and products were investigated by atomic force microscopy, which enabled atomic-scale visualization of the graphitic nitrogen sites. The electronic properties of the nanostructures were studied by scanning tunneling spectroscopy and density functional theory calculations. Our analyses revealed the presence of localized nitrogen-centered electronic states in the nanostructures. In the gas phase where the nanostructures are in the neutral charge state, these states undergo spin polarization leading to an open-shell ground state. Upon adsorption on Au(111), the nanostructures exhibit electron transfer to the surface, which resulted in a closed-shell ground state. Our results demonstrate a straightforward and generally applicable route to synthesize graphitic nitrogen-substituted carbon nanomaterials with potential applications in spintronics, catalysis, and energy storage.

## Graphical abstract

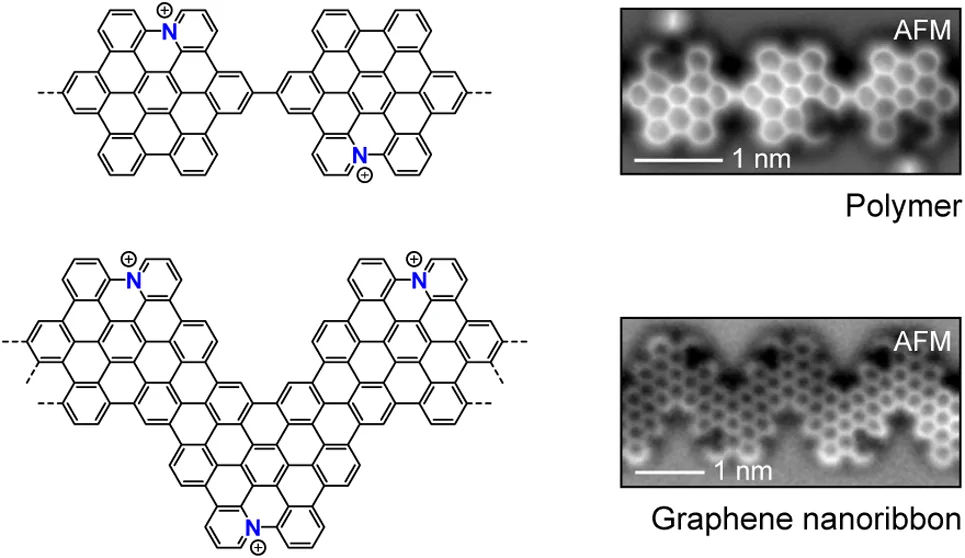

## Introduction

Heteroatom substitution offers a versatile approach to tailor the chemical and electronic properties of carbon nanomaterials. Nitrogen has been extensively studied as a substituent, particularly in the context of graphene. Following carbon in the periodic table, nitrogen shares characteristics such as a similar atomic radius and the possibility to exhibit 3-fold coordination with neighboring carbon atoms in the graphene lattice via sp^2^ hybridization.^1,2^ Owing to an additional valence electron in nitrogen compared to carbon, substitution of a 3-fold coordinated carbon atom with nitrogen in graphene (the substitutional nitrogen atom is referred to as graphitic nitrogen, denoted hereafter as gN) results in a shift of the Fermi level away from the Dirac point and leads to an n-type doping effect analogous to inorganic semiconductors.^3–5^ Beyond electronic transport, gN substitution in (nano)­graphene has also been explored as a route to imprint functionalities suitable for spintronics, catalysis, and energy storage.^6–10^

The proposed applications of gN-substituted carbon nanomaterials (particularly those pertaining to magnetism) require the periodic and deterministic placement of individual nitrogen atoms in carbon lattices. Conventional methods such as chemical vapor deposition and ion implantation inherently yield random distribution of nitrogen atoms and high defect densities.^11–13^ In this regard, on-surface synthesis provides a facile route toward the atomically precise placement of heteroatoms in carbon lattices.^14–16^ Recent works have shown the on-surface synthesis of polycyclic conjugated hydrocarbons and graphene nanoribbons (GNRs) containing gN, though many examples feature pyrazine moieties that consist of two nitrogen atoms at diametrically opposite vertices in a six-membered heterocycle, and whose properties differ from isolated gN atoms.^17–23^ The current literature on the on-surface synthesis of polycyclic frameworks with isolated gN atoms is mostly focused on model molecular systems, namely, aza-triangulene and derivatives,^24–28^ and mono-,^29^ di-^30^ and triaza-hexabenzocoronene (HBC) molecules.^31^ Consequently, existing works on gN-substituted GNRs are limited to three reports that also reveal the challenges of achieving long and defect-free GNRs with high fidelity of gN substitution. Gao et al. reported the on-surface synthesis of gN-substituted GNRs with partial corannulene motifs on Ag(100) via selective intramolecular activation of ortho C­(sp^3^)–H bonds in a phenanthridine derivative, followed by intermolecular polymerization.^32^ However, the competition between intramolecular cyclodehydrogenation and intermolecular polymerization, along with the competition between coupling of monomer units of the same or opposite chirality led to disordered polymer growth with short lengths. More recently, Zhang et al. reported the on-surface synthesis of fused aza-HBC GNRs on Au(111), wherein mostly short and disordered GNRs were obtained, and structural defects related to the loss of phenyl rings in the monomer units were frequently encountered.^29^ Finally, Bassi et al. recently reported the on-surface synthesis of modified armchair GNRs with repeating dibenzoovalene motifs. While the GNRs were long, approximately 10% of the repeating motifs exhibited pyridinic nitrogen (pN) substitution instead of the targeted gN substitution.^33^

In this work, we demonstrate the on-surface synthesis of one-dimensional carbon nanostructures with periodic gN substitution (where every nitrogen atom in a unit cell is a gN and every unit cell contains a predetermined number of nitrogen atoms) on Au(111) and their characterization at the atomic scale by scanning tunneling microscopy (STM), atomic force microscopy (AFM), and density functional theory (DFT) calculations. Specifically, we targeted the synthesis of a polymer and a chevron GNR based on covalently coupled HBC-like building blocks, each containing a single gN atom (Figure 1). Our work circumvents the issues encountered in previous reports on the growth of gN-substituted GNRs, namely, we achieve long and defect-free growth of gN-substituted polymers and GNRs, where each and every unit cell contains the expected number of gN atoms. In addition, we demonstrate that the dispersion of the frontier bands associated with gN substitution can be tuned through the structural design of the nanostructures, which has implications for spin and charge transport. We characterized the on-surface reaction steps and the bond-resolved chemical structures of the polymer (denoted as *poly*-gN-HBC) and the GNR (denoted as GNR-gN-HBC) by STM and AFM measurements at a temperature of 5 K. We probed the electronic properties of *poly*-gN-HBC and GNR-gN-HBC by scanning tunneling spectroscopy (STS) measurements. We detected and mapped the distribution of frontier electronic bands associated with gN substitution in real space (indicating substantial modification of the electronic properties of the gN-substituted nanostructures compared to the corresponding all-carbon counterparts) along with the bands associated with the carbon framework. In the gas phase, DFT calculations predicted an open-shell antiferromagnetic ground state of *poly*-gN-HBC and GNR-gN-HBC. However, comparison of STS measurements with DFT calculations indicated that each gN-HBC unit transferred an electron to Au(111), which resulted in a closed-shell ground state of *poly*-gN-HBC and GNR-gN-HBC in their cationic state.

**Figure 1:**
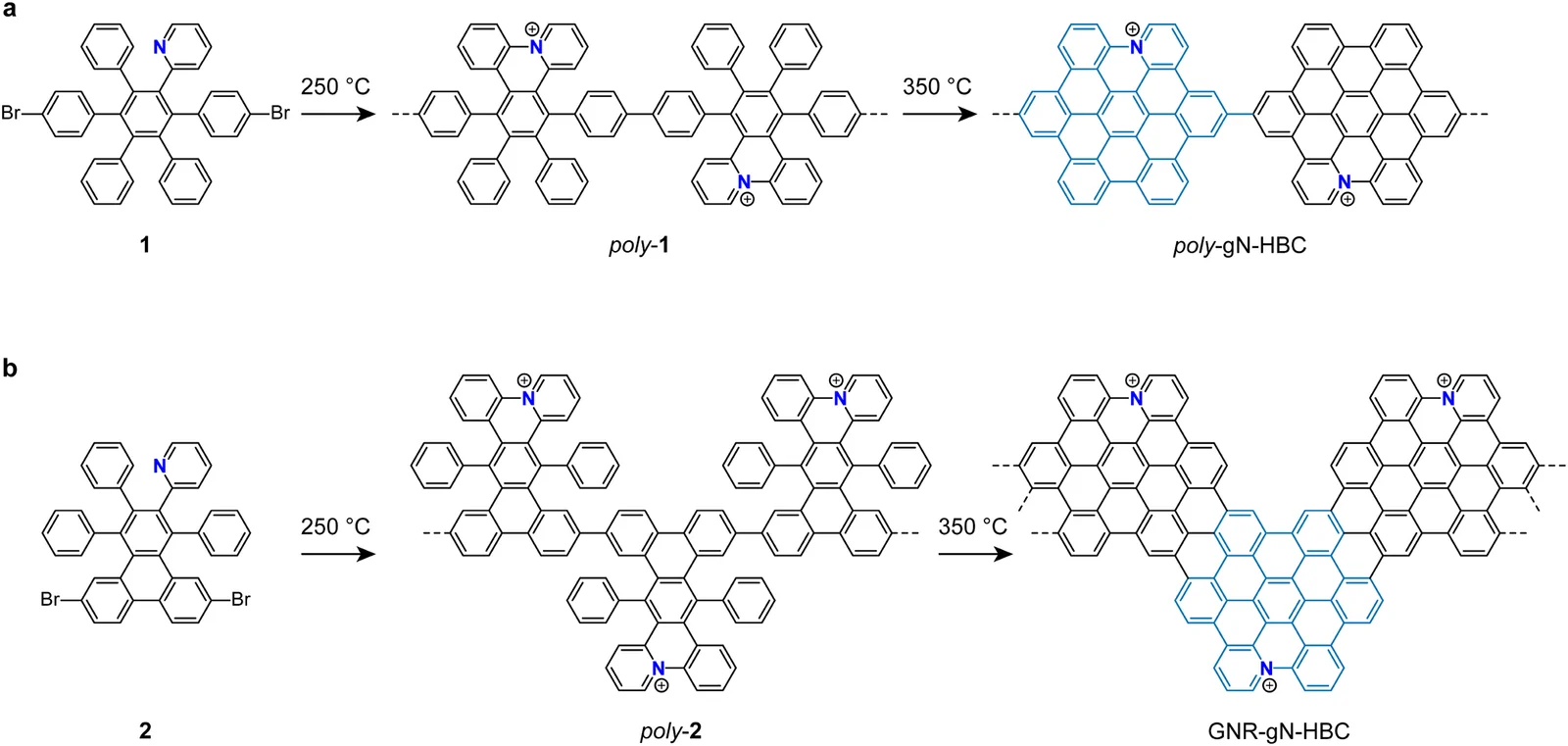
Synthetic route toward the graphitic nitrogen-substituted nanostructures reported in this work. (a, b) Scheme of on-surface synthesis of *poly*-gN-HBC (a) and GNR-gN-HBC (b) on Au(111). The gN-HBC units in *poly*-gN-HBC and GNR-gN-HBC are highlighted. We note that debrominative aryl–aryl coupling and C–N bond formation occur within different temperature windows, with our experiments suggesting that the former precedes the latter (see Figure S14). While we cannot specify the exact temperature windows within which the individual reactions occur, both reactions are nearly complete at 250 °C.

## Results and Discussion

### On-Surface Synthesis and Structural Characterization

Our strategy for synthesizing *poly*-gN-HBC and GNR-gN-HBC began with the solution-phase synthesis of the respective dibrominated precursors **1** and **2** (Figure 1; see Figures S1–S10 and accompanying text for details). Thereafter, submonolayer coverages of the precursors were deposited onto single-crystalline Au(111) surfaces held at room temperature and under ultrahigh vacuum (see Figures S11 and S12 for characterizations of the precursors). A two-step annealing first induced debrominative aryl–aryl coupling at ∼200 °C that led to intermediate polymer phases, followed by cyclodehydrogenation reaction at 350 °C that resulted in the formation of *poly*-gN-HBC and GNR-gN-HBC (shown schematically in Figure 1).^15^ Notably, we observed cyclodehydrogenative C–N bond formation to occur at temperatures around 200 °C, much lower than temperatures required for the corresponding C–C bond formation (typically occurring at 300 °C or above^34^) and in line with previous reports.^18,33^ Figures S13–S15 show scanning probe and theoretical characterizations of the intermediate polymer phases.

Figure 2a shows an STM overview image of the surface after a two-step annealing of **1** on Au(111) at temperatures of 250 and 350 °C, revealing the formation of *poly*-gN-HBC and highlighting the success of our synthetic strategy. High-resolution in-gap STM imaging of a *poly*-gN-HBC segment (Figure 2b) showed an overall smooth topography of the individual gN-HBC units. To directly visualize the gN sites, we conducted AFM imaging. Figure 2c shows an AFM image of a *poly*-gN-HBC segment, wherein the carbon framework of the gN-HBC units, the C–C bonds between the units, and a characteristic dark feature at the gN sites (in agreement with previous reports^30,35^ ) are evident (see Figure 2d for high-resolution AFM imaging of an individual gN-HBC unit, and Figure S16 for additional AFM data). Given the freedom of rotation of the pyridyl ring about the C–C single bond in **1**, C–N bond formation can occur either along the long axis of the polymer or at an angle of 60° to the long axis, which explains the varying positions of the nitrogen atoms in the segment. The simulated AFM image of a gN-HBC unit (Figure 2e) reproduced the features in the experimental AFM images, including the dark appearance of the gN sites.

**Figure 2:**
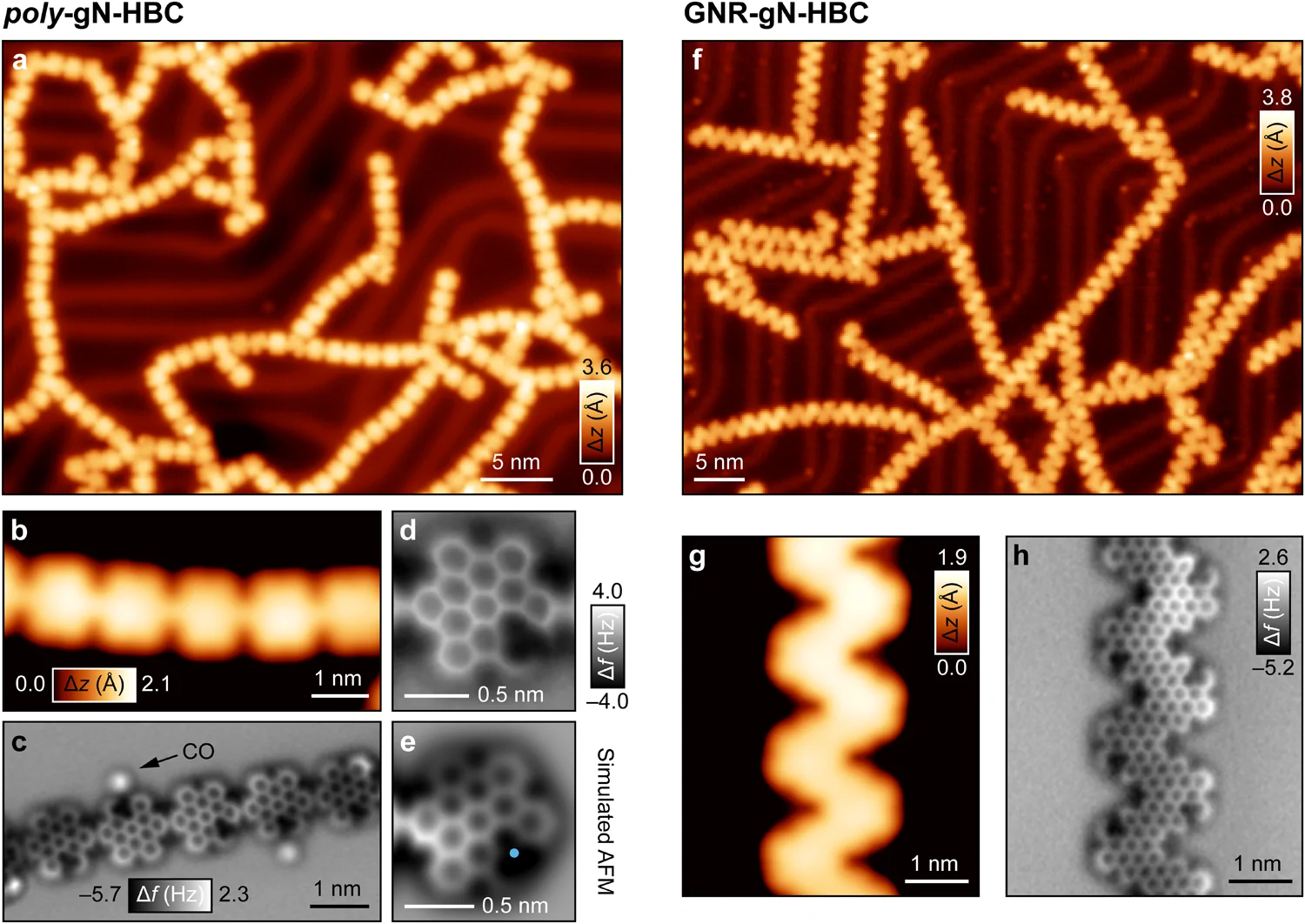
Imaging and structural characterization of the graphitic nitrogen-substituted nanostructures. (a) STM overview image after two-step annealing of **1** on Au(111) at 250 and 350 °C (*V* = −1.0 V, *I* = 100 pA). Δ*z* denotes the tip height, with positive (negative) values indicating tip retraction (approach) from the set-point conditions. (b) High-resolution STM image of a *poly*-gN-HBC segment (*V* = −30 mV, *I* = 120 pA). (c) AFM image of a *poly*-gN-HBC segment. Δ*f* denotes the frequency shift. A coadsorbed carbon monoxide (CO) molecule is indicated by an arrow. STM set-point: *V* = 5 mV and *I* = 100 pA on Au(111); Δ*z* = −0.3 Å. (d) High-resolution AFM image of an individual gN-HBC unit in a polymer. STM set-point: *V* = 5 mV and *I* = 100 pA on Au(111); Δ*z* = −0.5 Å. The images in (c, d) were acquired with the same tip. (e) Simulated AFM image of an individual gN-HBC unit in a gN-HBC dimer. The geometry of the dimer on Au(111) was optimized using DFT. The filled circle denotes the position of the nitrogen atom. (f) STM overview image after two-step annealing of **2** on Au(111) at 180 and 350 °C (*V* = −1.5 V, *I* = 30 pA). (g) High-resolution STM image of a GNR-gN-HBC segment (*V* = −0.2 V, *I* = 100 pA). (h) AFM image of the GNR-gN-HBC segment. STM set-point: *V* = 5 mV and *I* = 100 pA on Au(111); Δ*z* = −0.4 Å.

Similarly, we extended our protocol toward the synthesis of GNR-gN-HBC. Figure 2f,g shows overview and high-resolution STM images, respectively, after a two-step annealing of **2** on Au(111) at 180 and 350 °C, revealing the formation of nearly defect-free GNRs with maximum observed lengths of ∼50 nm. AFM imaging (Figure 2h) confirmed periodic gN incorporation in the GNRs, and similar to *poly*-gN-HBC, the GNRs exhibit both on- and off-axis C–N bond formation. The successful synthesis of both *poly*-gN-HBC and GNR-gN-HBC demonstrates the generality of our synthetic approach toward the formation of gN-substituted carbon nanostructures.

### Electronic Characterization

We first assessed the impact of nitrogen substitution on the electronic structure of all-carbon frameworks by DFT calculations, and we started by considering HBC as a model system. As shown in Figure 3a, the frontier electronic structure of HBC consists of a pair of degenerate orbitals constituting the highest occupied molecular orbital (HOMO) and HOMO–1, and a pair of degenerate orbitals constituting the lowest unoccupied molecular orbital (LUMO) and LUMO+1 (themselves nearly degenerate with the LUMO+2), along with a wide HOMO–LUMO gap of ∼2.50 eV. Replacing a =CH– group at the periphery by a nitrogen atom (pN) lifts the degeneracy of the frontier states and leads to a downward shift in the energies of the HOMO (LUMO) by 0.10 eV (0.22 eV) because of the higher electronegativity of nitrogen compared to carbon (Figure 3b).^36,37^ Related to nitrogen substitution in pN-HBC, there is a deep-lying nitrogen state (Figure 3b, indicated by a dashed ellipse). In contrast, gN substitution leads to a more drastic impact on the electronic structure. Whereas pN is isoelectronic to the =CH– group it replaces in a conjugated system, gN brings one more valence electron than the carbon atom it replaces. This additional valence electron leads to a nonbonding orbital in the gN-HBC spectrum (Figure 3c, left; indicated by a dashed ellipse). Inclusion of electronic correlations via spin-polarized DFT calculations leads to spin splitting of this state into singly occupied and corresponding unoccupied molecular orbitals (Figure 3c, right; also shown is the spin-density isosurface plot of the gN-HBC unit).

**Figure 3:**
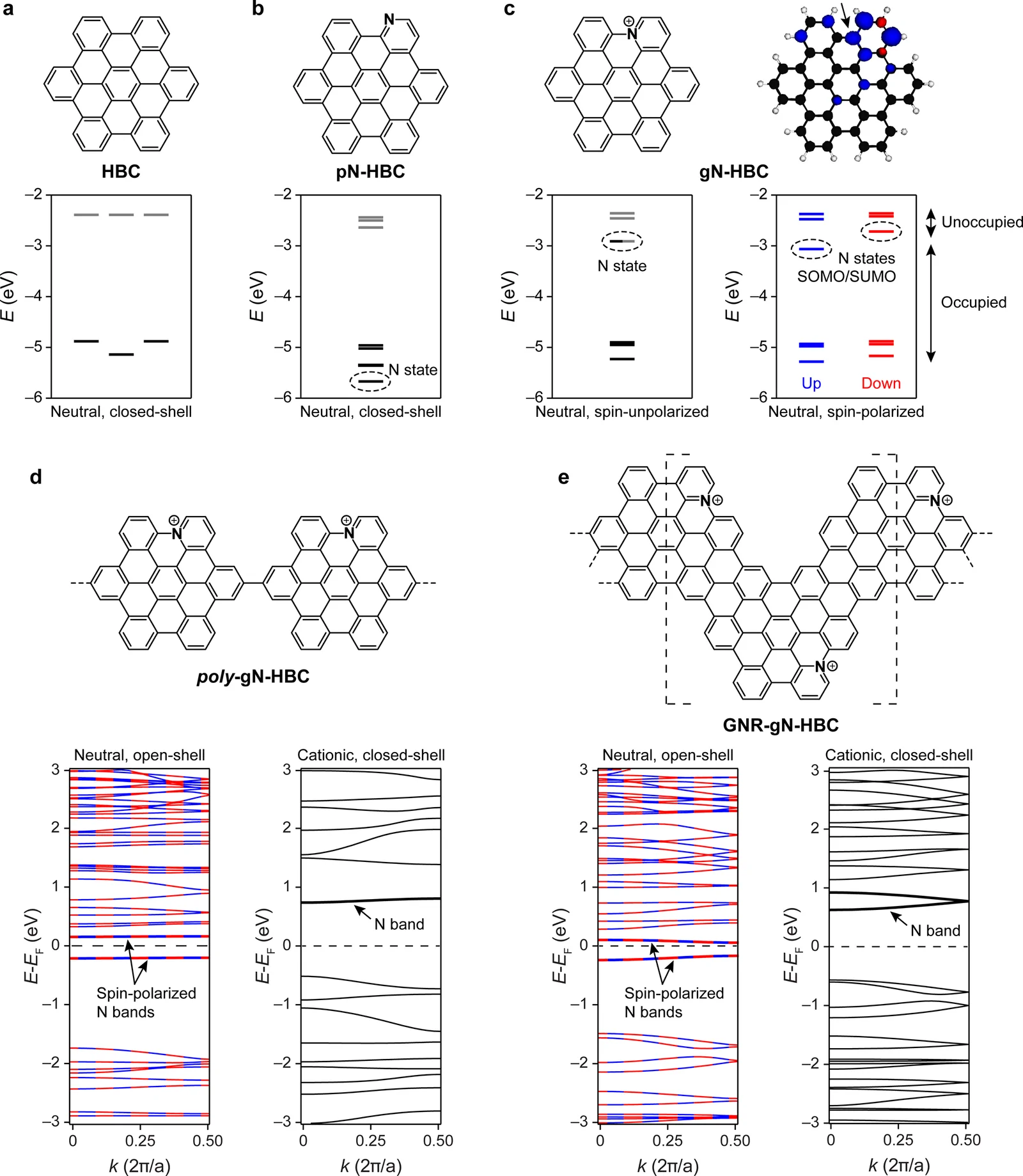
Theoretical electronic characterization of the graphitic nitrogen-substituted nanostructures. (a, b) Chemical structure and orbital energy diagram of HBC (a) and pN-HBC (b). Occupied and unoccupied states in the orbital energy diagrams in (a, b) and the left panel in (c) are colored black and gray, respectively. (c) Chemical structure and orbital energy diagram of neutral gN-HBC from a spin-unpolarized calculation (left), with the nonbonding nitrogen state indicated by a dashed ellipse. Also shown are the spin-density isosurface plot of gN-HBC (isovalue: 0.003 e/Bohr^3^; the location of the nitrogen atom is indicated by an arrow) and the orbital energy diagram from spin-polarized calculations (right). The two colors represent spin up and spin down. SOMO (SUMO) denotes singly occupied (unoccupied) molecular orbital. The energies in (a–c) are given relative to the vacuum level. (d, e) Chemical structure and band structure plots of *poly*-gN-HBC (d) and GNR-gN-HBC (e) in the neutral open-shell antiferromagnetic and cationic closed-shell states. In the open-shell state, spin-up and spin-down bands are degenerate. The energies in (d, e) are given relative to the respective Fermi energies (*E*_F_). The unit cells for the band structure calculations correspond to the gN-HBC monomer and dimer for the cationic and neutral states of *poly*-gN-HBC, respectively (d), and the area enclosed by the dashed brackets for GNR-gN-HBC (e).

By extension, in *poly*-gN-HBC, where the individual gN-HBC units are connected via C–C σ bonds, the weak electronic coupling between neighboring units gives rise to nearly dispersionless spin-polarized frontier bands associated with nitrogen substitution, and an antiferromagnetic coupling between neighboring units (Figure 3d, left). Incorporating gN atoms into the rigid π framework of GNR-gN-HBC leads to an increase in the dispersion of the spin-polarized frontier bands (Figure 3e, left) while keeping the ground state antiferromagnetic, which may make this system more suitable from a device point of view, particularly when spin and charge transport is desired. We note that the results of our calculations agree with a previous theoretical work by Azevêdo et al.^7^ Relative to the antiferromagnetic ground state, the energy of the ferromagnetic (closed-shell) state is 6 meV (130 meV) and 32 meV (62 meV) higher for *poly*-gN-HBC and GNR-gN-HBC, respectively (see Figure S17 for additional DFT calculations).

At the outset, we note that as opposed to their neutral charge states in the gas phase, both *poly*-gN-HBC and GNR-gN-HBC are positively charged (that is, in a cationic state) on Au(111) because of each constituent gN-HBC unit losing one electron to the surface. In such a scenario, *poly*-gN-HBC and GNR-gN-HBC are no longer open-shell systems as in the neutral charge state but are closed-shell. As shown in the DFT band structure calculations, the former singly occupied bands of both systems in the neutral state are emptied in the cationic state (Figure 3d,e, right). Consequently, the frontier bands in the cationic state correspond to a valence band (VB) with dominant contribution from the carbon atoms and a conduction band (CB) associated with nitrogen substitution.

Experimentally, we probed the electronic properties of *poly*-gN-HBC and GNR-gN-HBC by STS. Figure 4a shows d*I*/d*V* spectra (*I* and *V* denote the tunneling current and bias voltage, respectively) acquired at various locations on a *poly*-gN-HBC segment. The spectra exhibited four resonances at *V* = −1.85, 0.90, 1.45, and 1.80 V. To visualize the spatial distributions of the resonances, we acquired d*I*/d*V* maps at approximately these voltages (Figure 4b, top row) and compared them to the calculated local density of states (LDOS) maps of cationic *poly*-gN-HBC (Figure 4b, bottom row; corresponding bands are labeled in Figure 4c). The map at *V* = −1.85 V revealed a near-uniform distribution of intensities along the periphery of the gN-HBC units (the intensities are slightly modified by the presence of gN sites) and agreed well with the calculated LDOS map at the VB maximum with a dominant contribution from the carbon sites. The state at *V* = 0.90 V manifests as prominent lobes localized at the gN sites of the individual gN-HBC units and exhibited good agreement with the calculated LDOS map at the CB minimum, corresponding to the unoccupied nitrogen band. The states at *V* = 1.45 and 1.80 V, which are also distributed over the periphery of the gN-HBC units, are assigned to the CB+1 and CB+2, respectively, with dominant contributions from the carbon sites (see Figure S18 for additional d*I*/d*V* maps).

**Figure 4:**
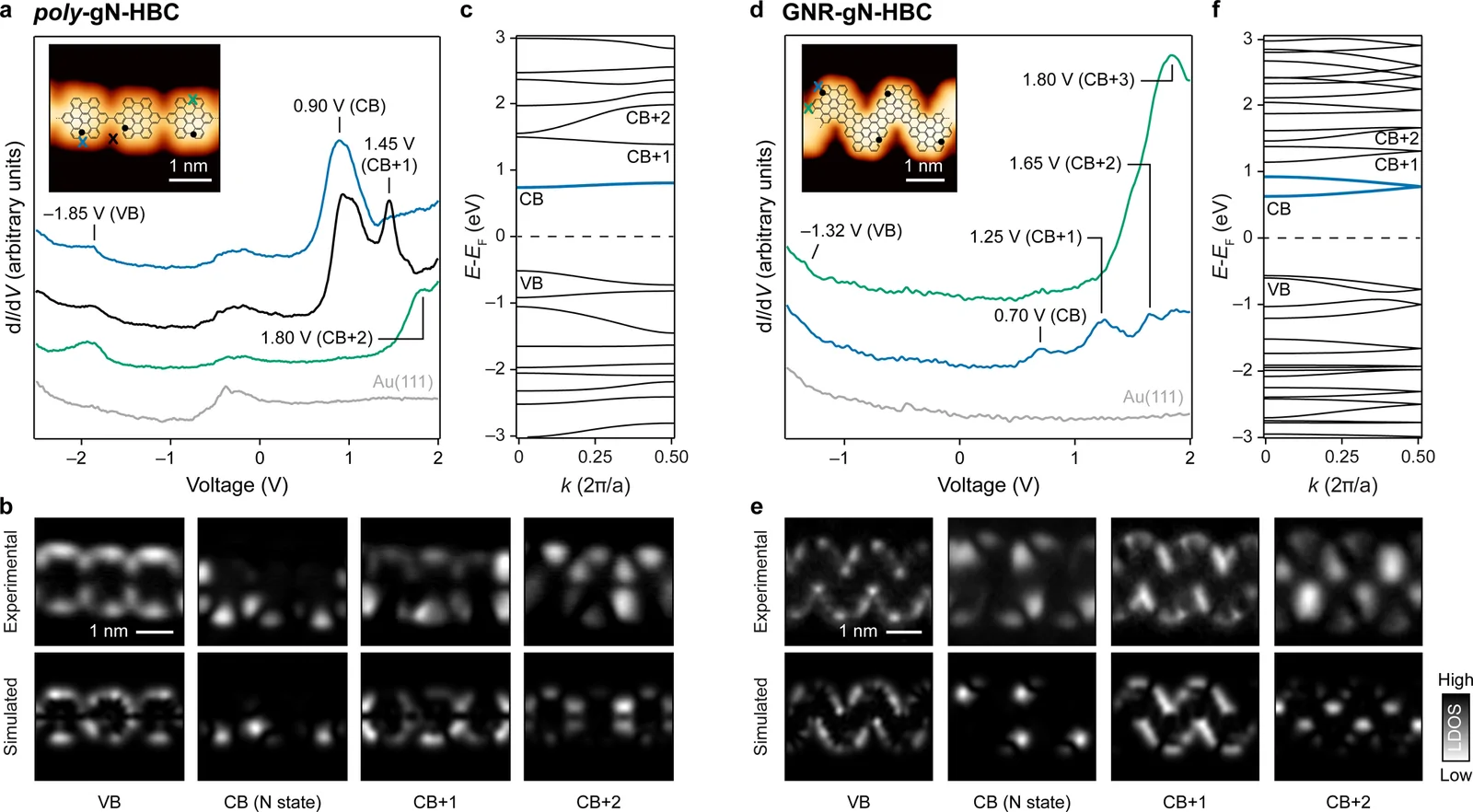
Experimental electronic characterization of the graphitic nitrogen-substituted nanostructures. (a) d*I*/d*V* spectra acquired on a *poly*-gN-HBC segment. Open feedback parameters: *V* = −2.50 V and *I* = 200 pA on the individual units; root-mean-square modulation voltage in the lock-in amplifier *V*_rms_ = 16 mV. Acquisition positions of the spectra are indicated by the respective colored crosses in the inset STM image (*V* = −30 mV, *I* = 120 pA). The chemical structures of the *poly*-gN-HBC and GNR-gN-HBC segments are superimposed on the STM images in (a, d), respectively; filled circles denote the positions of the nitrogen atoms. (b) Constant-current d*I*/d*V* maps of the segment acquired at voltages corresponding to the resonances in (a) (top) and the corresponding simulated LDOS maps (bottom). Scanning parameters (from left to right): *V* = −1.85 V, *I* = 250 pA; *V* = 0.70 V, *I* = 200 pA; *V* = 1.44 V, *I* = 200 pA; and *V* = 1.82 V, *I* = 200 pA; *V*_rms_ = 10 mV. (c) Band structure of cationic *poly*-gN-HBC, with the bands relevant to the STS measurements labeled. (d) d*I*/d*V* spectra acquired on a GNR-gN-HBC segment. Open feedback parameters: *V* = −2.00 V and *I* = 200 pA (*I* = 300 pA for the blue curve); *V*_rms_ = 20 mV. Acquisition positions are indicated by the respective colored crosses in the inset STM image (*V* = −0.20 V, *I* = 100 pA). (e) Constant-current d*I*/d*V* maps of the segment acquired at voltages corresponding to the resonances in (d) (top), and the corresponding simulated LDOS maps (bottom). Scanning parameters (from left to right): *V* = −1.35 V, *I* = 200 pA; *V* = 0.70 V, *I* = 200 pA; *V* = 1.20 V, *I* = 200 pA; and *V* = 1.60 V, *I* = 200 pA; *V*_rms_ = 10 mV. (f) Band structure of cationic GNR-gN-HBC, with the bands relevant to the STS measurements labeled. The unoccupied nitrogen bands (CB) are highlighted in color and with a thicker line stroke in (c, f).

d*I*/d*V* spectra acquired on a GNR-gN-HBC segment (Figure 4d) exhibited four frontier resonances at *V* = −1.32, 0.70, 1.25, and 1.65 V (note that the feature at −1.32 V, which is only visible as a small bump in the d*I*/d*V* spectrum due to a large contribution from tip DOS, is better resolved via d*I*/d*V* mapping). The corresponding d*I*/d*V* maps (Figure 4e, top row) revealed that the state at *V* = −1.32 V is distributed over the periphery of the GNR with enhanced intensity at the gN sites, which agreed well with the calculated LDOS map (Figure 4e, bottom row; see Figure 4f for the corresponding bands) at the VB maximum, which has a dominant contribution from the carbon sites. The state at *V* = 0.70 V is localized at the gN sites and agreed well with the calculated LDOS map at the CB minimum, corresponding to the unoccupied nitrogen band. Similarly, the states at *V* = 1.25 V, distributed over the GNR periphery with particularly strong intensities along the non-nitrogen-containing oblique edges, and at *V* = 1.65 V, with localized intensities at the bay regions, correspond to the CB+1 and CB+2 of the GNR, respectively, with dominant contributions from the carbon sites. The enhanced delocalization due to the rigid π framework of the GNR manifested in the nitrogen state in GNR-gN-HBC (*V* = 0.70 V) being closer to the Fermi energy than in *poly*-gN-HBC (*V* = 0.90 V).

## Conclusions

In summary, we demonstrated the on-surface synthesis of two graphitic nitrogen-substituted one-dimensional carbon nanostructures, a polymer, and a graphene nanoribbon, with atomic precision and periodic nitrogen substitution. The success of our synthetic protocol, along with the high fidelity of nitrogen substitution in the nanostructures, is demonstrated via atomic-scale imaging using scanning probe techniques. Spectroscopic measurements, in combination with density functional theory calculations, revealed localized nitrogen-centered bands. In the neutral charge state of the nanostructures, the nitrogen-centered bands are spin-polarized with an antiferromagnetic coupling between neighboring nitrogen sites. We also demonstrated that the dispersion of the nitrogen-centered bands, which can influence transport, can be controlled by the nanostructure design. Our synthetic protocol can be extended to graphitic nitrogen incorporation in a range of carbon nanostructures with different structural designs and dimensionalities. This provides a route to imprint magnetism and energy-storage functionalities in organic materials. A current limitation that we encounter is that the exact site of nitrogen incorporation in the monomer units cannot be chosen, and future works could explore appropriate precursor designs that would allow the synthesis of nanostructures with a predetermined sequence of nitrogen incorporation. This could enable controllable electronic coupling between nitrogen sites for band structure engineering.

## Methods

### Sample Preparation and Scanning Probe Characterization

Au­(111) single-crystal surfaces (MaTeck GmbH) were prepared by iterative cycles of Ar^+^ sputtering and annealing at 723 K. Prior to the sublimation of molecules, the surface structure and cleanliness were checked by STM imaging. Powder samples of **1** and **2** were contained in quartz crucibles and sublimed from a home-built evaporator at 473 K (**1**) and 503 K (**2**) onto the single-crystal surfaces held at room temperature.

STM and AFM measurements were performed with two instruments from Scienta Omicron operated at a temperature of 5 K and base pressures below 5 × 10^–11^ mbar. Bias voltages were applied to the sample with respect to the tip. AFM measurements were performed in noncontact mode with a qPlus sensor.^38^ The sensor was operated in frequency-modulation mode^39^ with a constant oscillation amplitude of 0.5 Å. STM images were acquired in constant-current mode, and AFM images were acquired in constant-height mode with CO-functionalized tips at *V* = 5 mV. d*I*/d*V* spectra were acquired in constant-height mode, and d*I*/d*V* maps were acquired in both constant-height and constant-current mode using lock-in amplifiers (SR830 from Stanford Research Systems operating at a frequency of 860 Hz, and HF2Li from Zurich Instruments operating at 691 Hz). STM and AFM images, and d*I*/d*V* spectra and maps, were postprocessed using Gaussian low-pass filters in WaveMetrics Igor Pro software.

### Density Functional Theory Calculations

DFT calculations were executed using the AiiDAlab^40,41^ applications, based on AiiDA^42^ work chains designed for the DFT codes CP2K^43^ (systems adsorbed on gold) and Quantum Espresso (band structure calculations).^44^ Surface–adsorbate setups were modeled within a periodic slab scheme. The simulation cell included four gold atomic planes along the [111] orientation. Hydrogen atoms passivated one face of the slab to suppress the Au(111) surface state. A 40 Å vacuum layer was included to isolate the system from its periodic images along the axis orthogonal to the slab. Electronic wave functions were represented via the TZV2P Gaussians basis sets for carbon, nitrogen and hydrogen atoms, and the DZVP basis set for gold atoms. Plane-waves basis set cutoff for the charge density was set at 600 Ry. Norm-conserving Goedecker–Teter–Hutter pseudopotentials were employed. The PBE GGA^45^ approximation for the exchange correlation functional was used and Grimme’s D3 ^46^ van der Waals corrections were included. Gold supercells varied in size depending on the adsorbate, ranging from 28.12 Å × 26.54 Å (corresponding to 432 gold atoms) to 23.60 Å × 40.85 Å (corresponding to 648 gold atoms). Geometry optimizations were performed with the bottom two atomic planes constrained while relaxing others, until forces were below 0.005 eV Å^–1^. For AFM simulations, DFT equilibrium geometries and electrostatic potentials were used alongside the probe-particle model.^47^

For the band structure calculations, ultrasoft pseudopotentials from the SSSP^49^ were employed to model the ionic potentials. A cutoff of 50 Ry (400 Ry) was used for the plane-wave expansion of the wave functions (charge density). The simulation cell contained 15 Å of vacuum in the nonperiodic directions to minimize interactions among periodic replicas of the system. The thickness of the vacuum region, the sampling of the Brillouin zone and the cutoff ensure convergence of the computed band structures. The atomic positions of the polymer and GNR atoms, and the cell dimension along the ribbon axis were optimized until forces were lower than 0.002 eV Å^–1^ and the pressure in the cell was negligible.

## Supplementary Material



## Notes

Solution synthesis and characterization of precursor molecules **1** and **2**, scanning probe measurements of precursor, intermediate polymer phases, *poly*-gN-HBC and GNR-gN-HBC, and DFT calculations (PDF)
