# Supplementary material for: One-Dimensional Carbon Nanostructures with Periodic Graphitic Nitrogen Substitution
Source: ACS Nano. 2026 Oct 30;20(44):29869–77. doi: 10.1021/acsnano.6c12638 (PMC13758423; doi:10.1021/acsnano.6c12638)
Supplement: Supplementary file 1 [file nn6c12638_si_001.pdf]

Supporting Information: One-dimensional carbon nanostructures with periodic graphitic nitrogen substitution

Nicolò Bassi,[†,#] Shantanu Mishra,[*,†,‡,#] Zheng Zhang,[¶] Xiao-Ye Wang,[§,‖] Feifei Xiang,[†] Nils Krane,[†] Carlo A. Pignedoli,[†] Klaus Müllen,[§] Pascal Ruffieux,[*,†] Akimitsu Narita,[*,¶,§] and Roman Fasel[†,⊥]

†*nanotech@surfaces Laboratory, Empa—Swiss Federal Laboratories for Materials Science and Technology, 8600 Dübendorf, Switzerland*

‡*Department of Physics and Astronomy, Chalmers University of Technology, 412 96 Göteborg, Sweden*

¶*Organic and Carbon Nanomaterials Unit, Okinawa Institute of Science and Technology Graduate University, 904-0495 Okinawa, Japan*

§*Max Planck Institute for Polymer Research, 55128 Mainz, Germany*

‖*College of Chemistry, Nankai University, 300071 Tianjin, P.R. China*

⊥*Department of Chemistry, Biochemistry and Pharmaceutical Sciences, University of Bern, 3012 Bern, Switzerland*

#*These authors contributed equally to this work*

E-mail: shantanu.mishra@chalmers.se; pascal.ruffieux@empa.ch; akimitsu.narita@oist.jp

Contents:

Solution-phase synthesis and characterization 3

Scanning probe measurements and calculations 11

References 19

Solution-phase synthesis and characterization

All reactions working with air- or moisture-sensitive compounds were carried out under an argon atmosphere using standard Schlenk line techniques. 5,10-Dibromo-1,3-diphenyl-2H-cyclopenta[*l*]phenanthren-2-one (**S6**) was prepared by adapting previously reported synthetic procedures.[1] Unless otherwise noted, all starting materials and other chemicals were purchased from commercial sources and used without further purification. Thin layer chromatography (TLC) was done on silica gel coated aluminum sheets with F254 indicator and column chromatography separation was performed with silica gel (particle size 0.040-0.063 mm). Nuclear Magnetic Resonance (NMR) spectra were recorded using JEOL JNM-ECA 600 MHz NMR spectrometers. Chemical shifts (δ) were expressed in parts per million (ppm) relative to the residual solvent ($CDCl_3$, ^{1}H: 7.26 ppm, ^{13}C: 77.16 ppm). Coupling constants (J) were recorded in Hertz. High-resolution mass spectra (HRMS) were recorded on a Bruker UltrafleXtreme spectrometer by matrix-assisted laser decomposition/ionization (MALDI) using *trans*-2-[3-(4-*tert*-butylphenyl)-2-methyl-2-propenylidene]malononitrile (DCTB) as matrix and calibrating with fleXstandard polymers.

2-(Phenylethynyl)pyridine (**S3**)

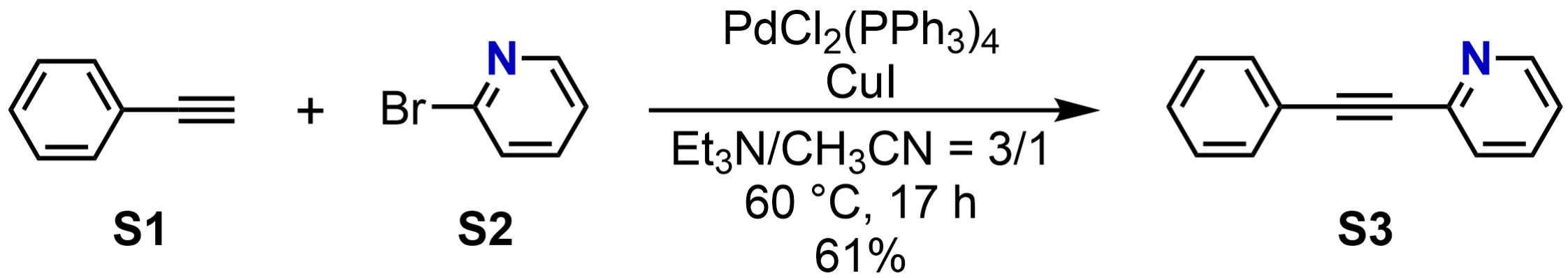

Figure S1: Synthesis of compound **S3**

In a 100-mL two-neck flask, a solution of ethynylbenzene (**S1**) (3.4 mL, 28 mmol) and 2-bromopyridine (**S2**) (3.0 mL, 31 mmol) in triethylamine (30 mL) and acetonitrile (10 mL) was prepared and purged with argon by bubbling for 30 min. CuI (0.61 g, 3.2 mmol) and $PdCl_2(PPh_3)_4$ (2.2 g, 3.2 mmol) were then added, and the reaction mixture was stirred at 60 °C using a sea sand bath for 17 h under argon atmosphere. After cooling down to room temperature, the mixture was extracted with dichloromethane (100 mL × 3) and washed once with brine (100 mL × 1). The organic layer was dried over $MgSO_4$, filtered, and concentrated under reduced pressure. The crude product was purified by column chromatography on silica gel (eluent: hexane/ethyl acetate = 4/1), and the removal of the solvents under reduced pressure afforded compound S3 as a light-yellow solid (3.0 g, 17 mmol, 61%). The ^{1}H NMR spectrum is shown in Figure S1, which is consistent with a previous report.[2]

^{1}H NMR (600 MHz, $CDCl_3$, 298 K): δ 8.63 (s, 1H), 7.68 (td, $J_1 = 7.7$ Hz, $J_2 = 0.9$ Hz, 1H), 7.62–7.58 (br, 2H), 7.53 (d, $J = 7.8$ Hz, 1H), 7.39–7.34 (br, 3H), 7.24 (ddd, $J_1 = 7.6$ Hz, $J_2 = 4.9$ Hz, $J_3 = 1.0$ Hz, 1H).

1-(Pyridin-2-yl)-2,4,5-tris(phenyl)-3,6-bis(4-bromophenyl)benzene (**1**)

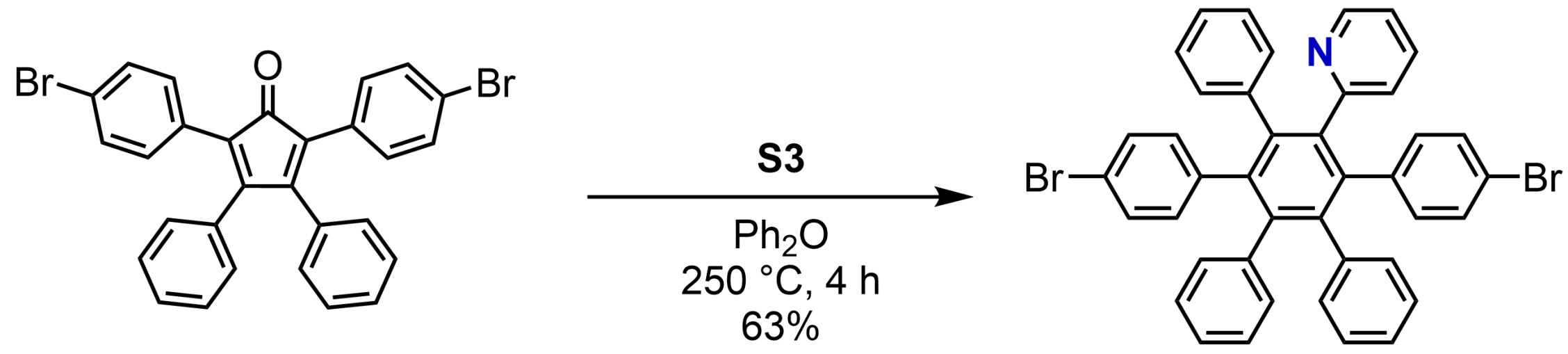

Figure S2: Synthesis of compound **1**

In a 50-mL two-neck flask, a solution of compound **S3** (360.8 mg, 2.013 mmol) and 2,5-bis(4-bromophenyl)-3,4-diphenylcyclopenta-2,4-dienone (**S4**) (1.006 g, 1.855 mmol) in diphenyl ether (10 mL) was placed. The reaction vessel was purged with argon and vacuum three times to create an inert atmosphere, and then the mixture was stirred at 250 °C for 4 h using a sand bath under an argon atmosphere. After the solution was cooled to room temperature, the crude mixture was purified by column chromatography on silica gel (eluent: dichloromethane) to afford compound **1** as a white solid (814.0 mg, 1.174 mmol, 63%).

^{1}H NMR (600 MHz, $CDCl_3$, 298 K): δ 8.20 (ddd, J_1 = 4.9 Hz, J_2 = 1.8 Hz, J_3 = 1.0 Hz, 1H), 7.21 (td, J_1 = 7.7 Hz, J_2 = 0.9 Hz, 1H), 6.98 (dd, J_1 = 7.4 Hz, J_2 = 1.4 Hz, 4H), 6.88–6.66 (br, 21H).

^{13}C{^{1}H} NMR (151 MHz, $CDCl_3$, 298 K): δ 159.12, 148.36, 141.45, 140.67, 140.21, 140.05, 139.87, 139.85, 139.73, 139.58, 139.31, 139.20, 134.79, 133.23, 133.13, 133.10, 133.07, 133.03, 132.98, 132.95, 132.33, 132.18, 131.93, 131.44, 131.34, 131.32, 131.24, 130.83, 130.73, 130.06, 130.02, 129.99, 129.95, 127.05, 126.99, 126.51, 125.83, 125.77, 120.72, 119.85, 119.78.

HRMS (MALDI–TOF, positive) (m/z): $[M+H]^+$ calcd for $C_{41}H_{28}Br_2N^+$, 692.0583; found, 692.0556.

5,10-Dibromo-1,3-diphenyl-2H-cyclopenta[*l*]phenanthren-2-one (**S6**)[2]

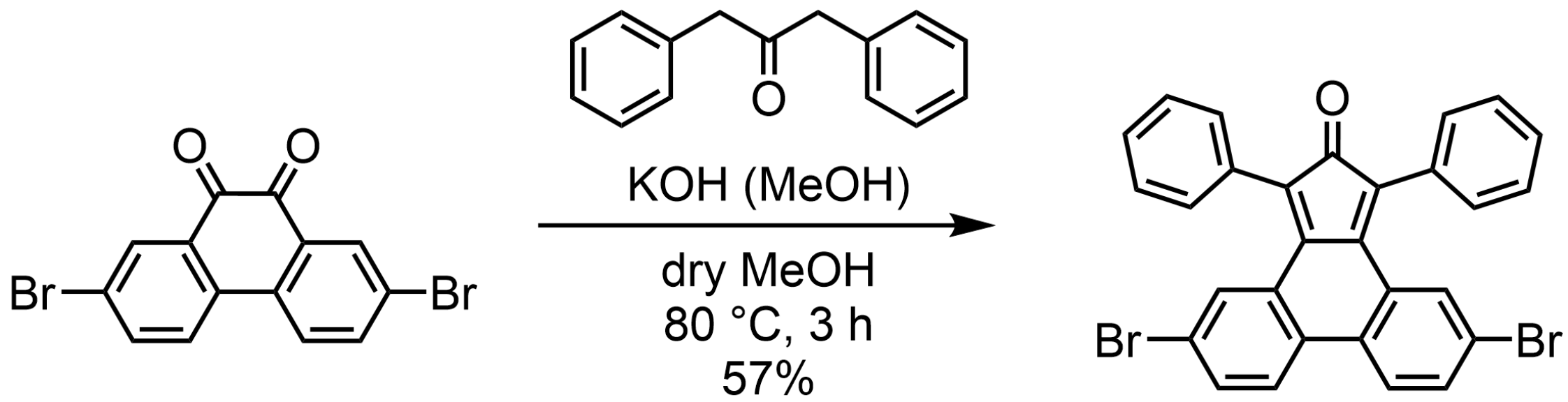

S5

S6

Figure S3: Synthesis of compound **S6**

In a 100-mL two-neck flask, a solution of 2,7-dibromophenanthrene-9,10-dione (**S5**) (2.671 g, 7.298 mmol) and 1,3-diphenyl-2-propanone (1.892 g, 8.998 mmol) in dry methanol (15 mL) was placed. The reaction mixture was stirred at 80 °C using a sea sand bath under argon atmosphere. A solution of KOH (431.6 mg, 7.692 mmol) in dry methanol (25 mL) was added dropwise over 1 h via a dropping funnel, and the resulting mixture was stirred for an additional 2 h at the same temperature. After cooling down to room temperature, the mixture was filtered, and the collected solid was washed with toluene (20 mL × 5) to afford compound **S2** as a deep green solid (2.263 g, 4.189 mmol, 57%). The ^{1}H NMR spectrum is shown in Figure S4, which is consistent with a previous report.[1]

^{1}H NMR (600 MHz, $CDCl_3$, 298 K): δ 7.66 (d, J = 2.0 Hz, 2H), 7.61 (d, J = 8.7 Hz, 2H), 7.48–7.36 (br, 12H).

10-(Pyridin-2-yl)-9,11,12-tris(phenyl)-2,7-dibromotriphrnylene (**2**)

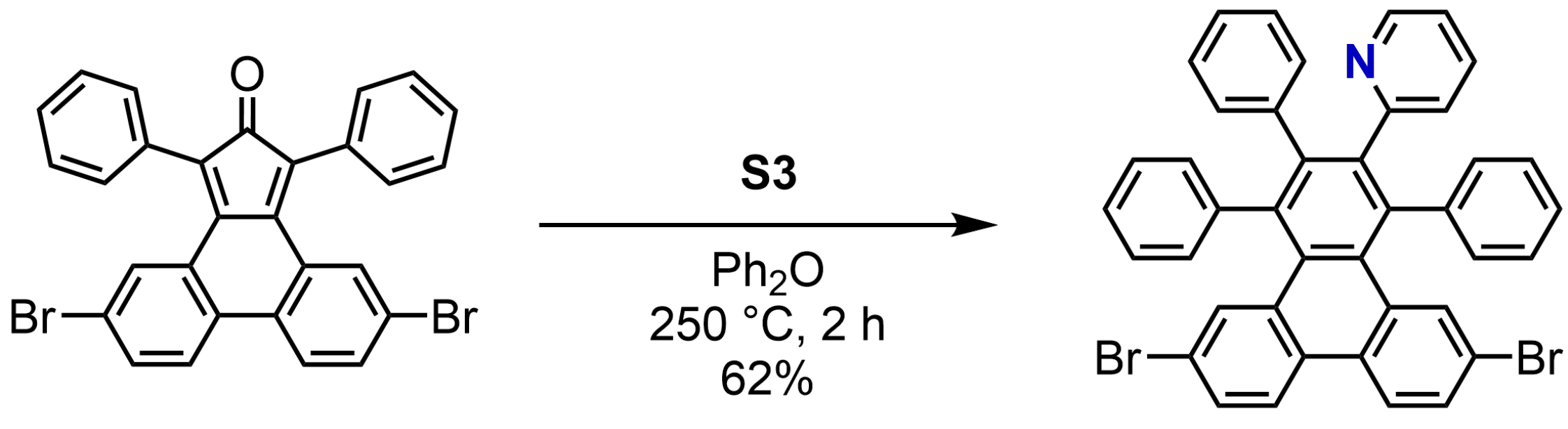

S6 **2**

Figure S4: Synthesis of compound **2**

In a 50-mL two-neck flask, a solution of compound **S3** (378.4 mg, 2.111 mmol) and compound **S6** (1.016 g, 1.881 mmol) in diphenyl ether (10 mL) was placed. The reaction vessel was purged with argon and vacuum three times to create an inert atmosphere, and then the mixture was stirred at 250 °C for 2 h using a sand bath under an argon atmosphere. After the solution was cooled to a room temperature, the reaction mixture was purified by column chromatography on silica gel (eluent: dichloromethane) to afford compound **2** as a slightly yellowish white solid (804.1 mg, 1.163 mmol, 62%).

^{1}H NMR (600 MHz, $CDCl_3$, 298 K): δ 8.22–8.20 (br, 3H). 7.69 (d, J = 2.0 Hz, 1H), 7.67 (d, J = 2.0 Hz, 1H), 7.49 (ddd, J_1 = 8.7 Hz, J_2 = 1.7 Hz, J_3 = 1.7 Hz, 2H), 7.24 (td, J_1 = 7.7 Hz, J_2 = 0.9 Hz, 1H), 7.20–7.14 (br, 7H), 7.06–7.04 (br, 3H), 6.94–6.88 (br, 3H), 6.84 (ddd, J_1 = 7.6 Hz, J_2 = 4.9 Hz, J_3 = 1.1 Hz, 1H), 6.80–6.78 (br, 2H), 6.69–6.68 (br, 1H).

^{13}C{^{1}H} NMR (151 MHz, $CDCl_3$, 298 K): δ 159.15, 148.28, 141.92, 141.80, 141.74, 140.90, 140.56, 139.75, 137.96, 137.61, 134.55, 134.50, 132.98, 132.34, 132.31, 132.03, 131.98, 131.81, 131.55, 131.07, 131.00, 130.72, 130.35, 129.83, 129.77, 129.74, 129.70, 128.70, 128.59, 128.48, 128.41, 127.09, 127.05, 127.02, 126.99, 126.86, 125.70, 124.60, 120.65, 120.27, 120.25.

HRMS (MALDI–TOF, positive) (m/z): $[M+H]^+$ calcd for $C_{41}H_{26}Br_2N^+$, 690.0427; found, 690.0460.

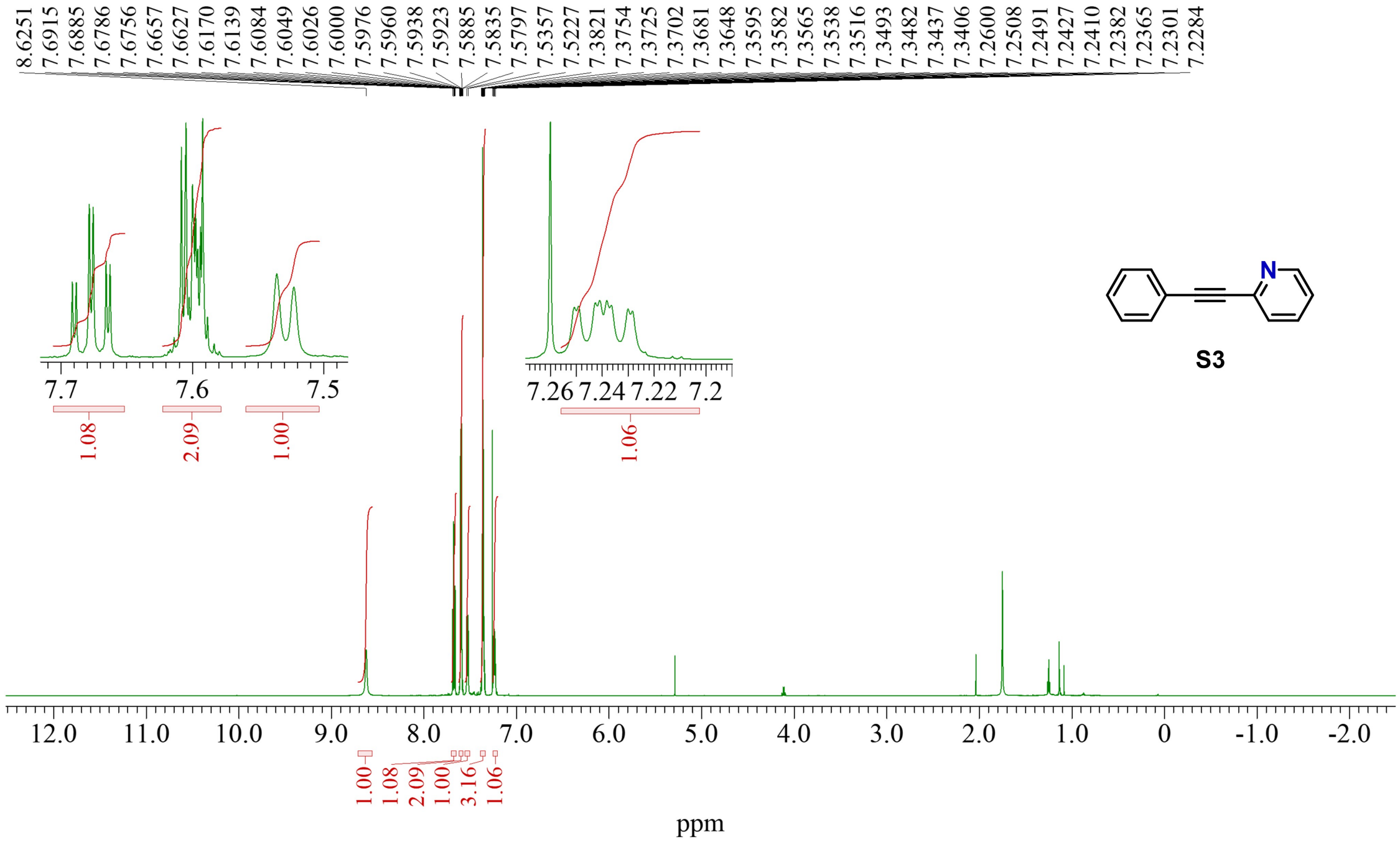

Figure S5: ^{1}H NMR spectrum of compound **S3** ($CDCl_3$, 600 MHz, 298 K).

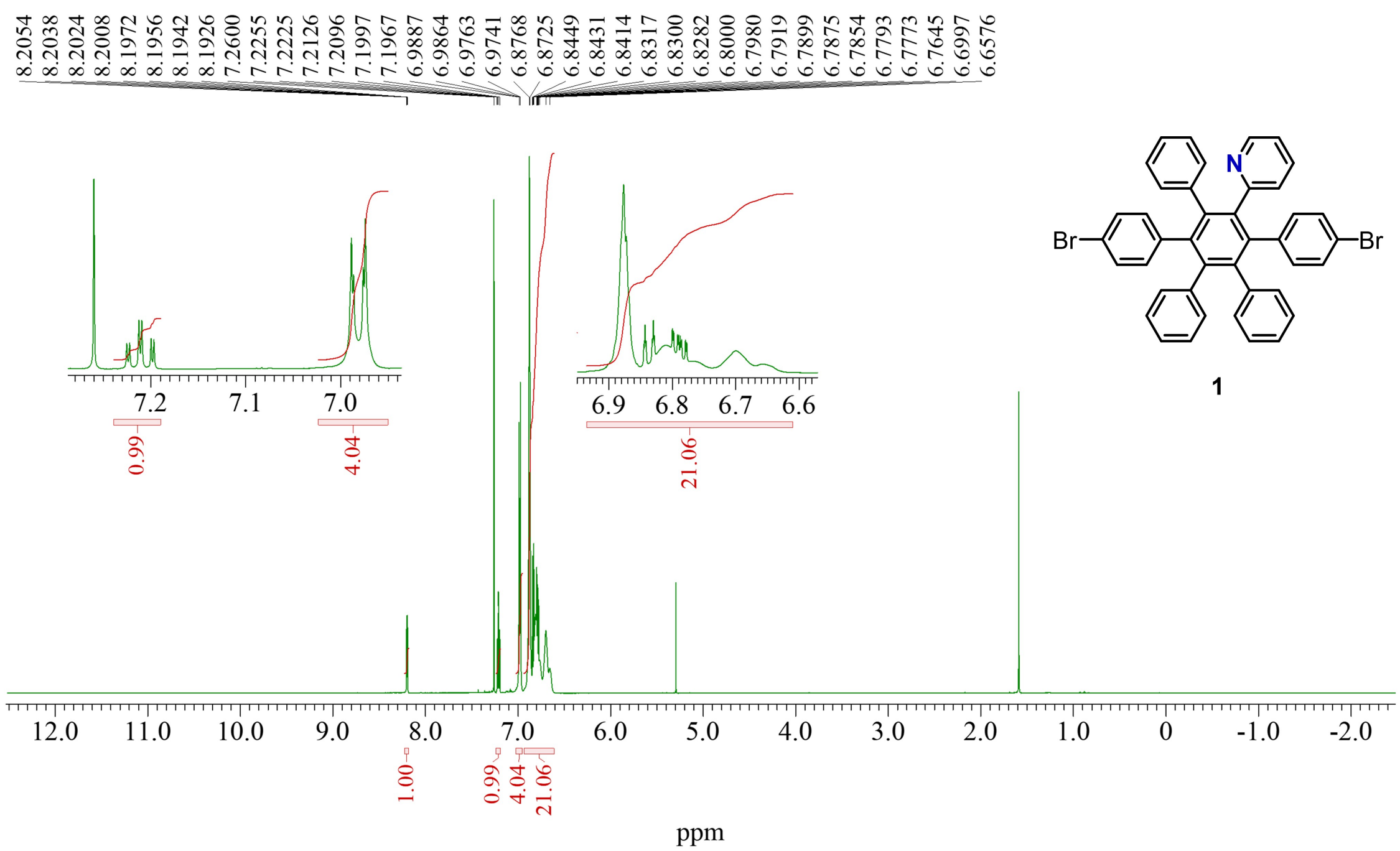

Figure S6: ^{1}H NMR spectrum of compound **1** ($CDCl_3$, 600 MHz, 298 K).

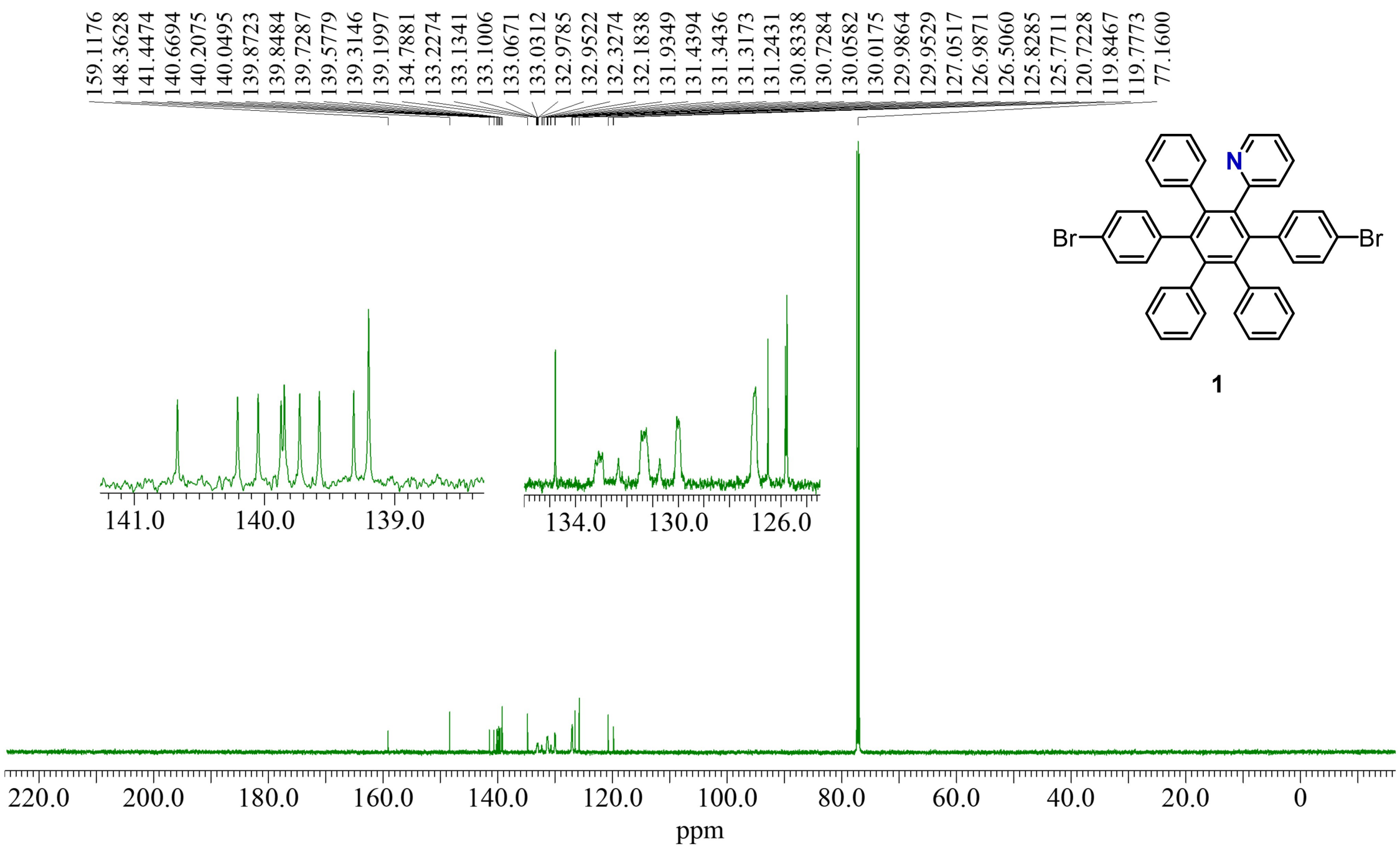

Figure S7: ^{13}C NMR spectrum of compound **1** ($CDCl_3$, 126 MHz, 298 K).

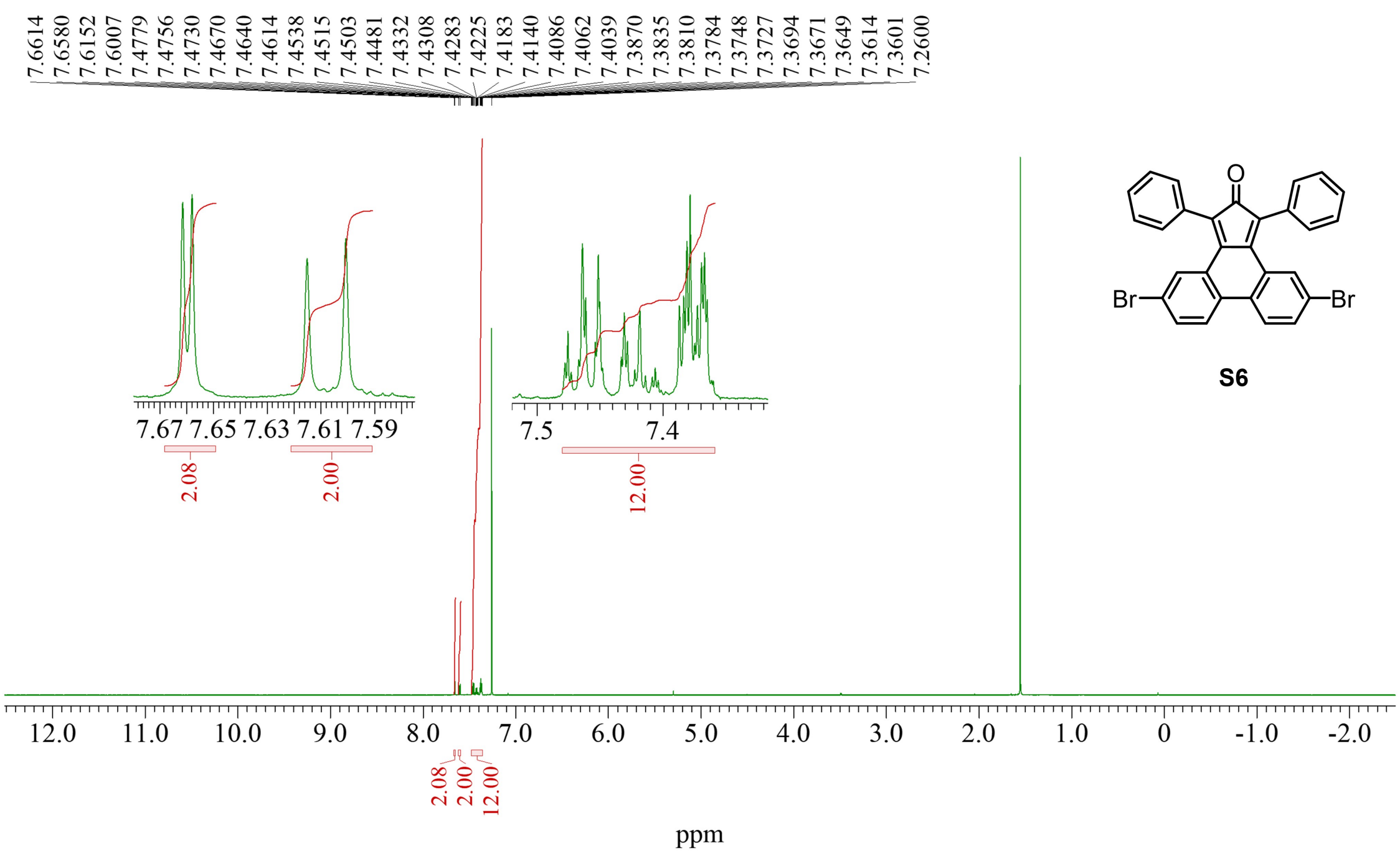

Figure S8: ^{1}H NMR spectrum of compound **S6** ($CDCl_3$, 600 MHz, 298 K).

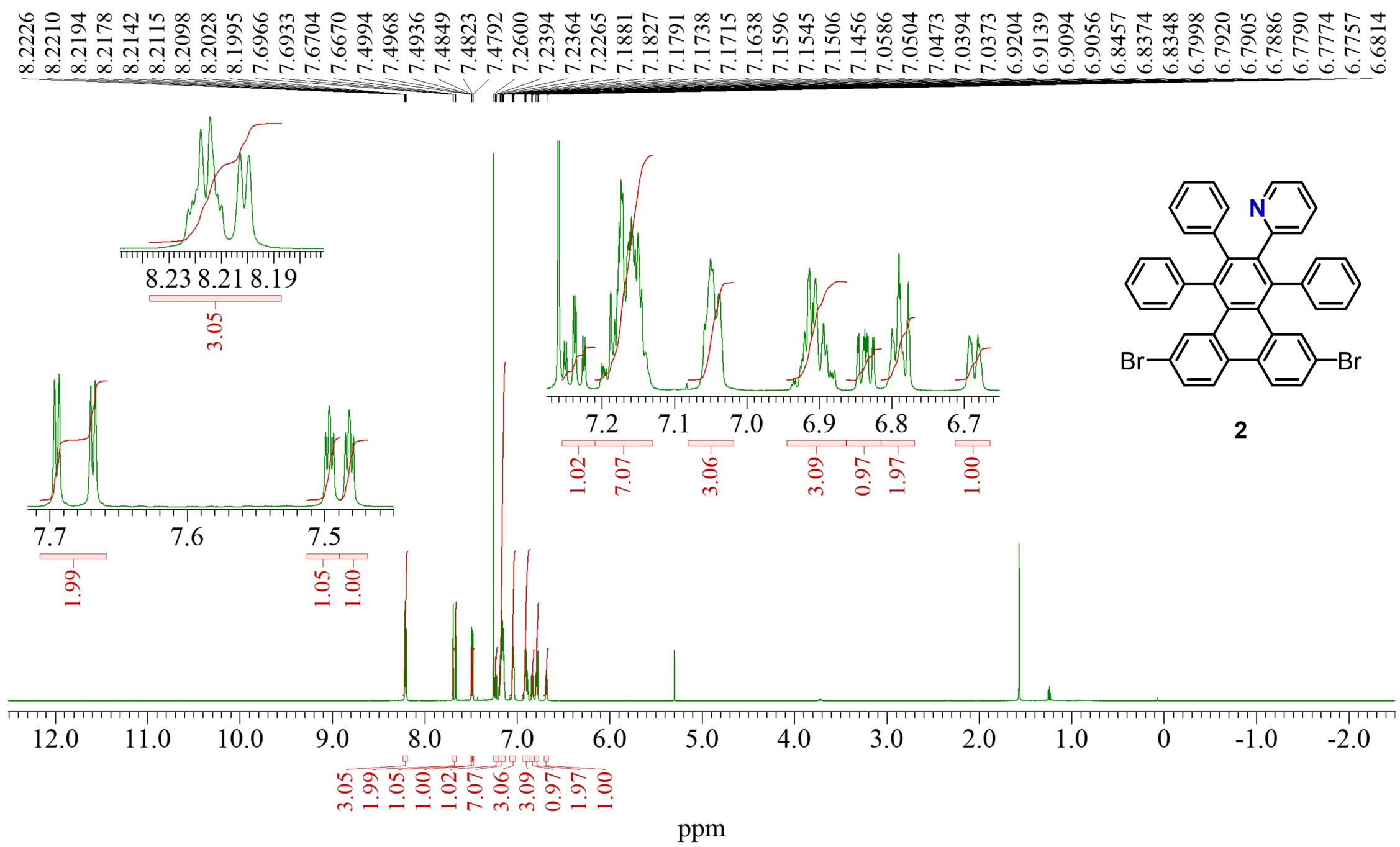

Figure S9: ^{1}H NMR spectrum of compound **2** ($CDCl_3$, 600 MHz, 298 K).

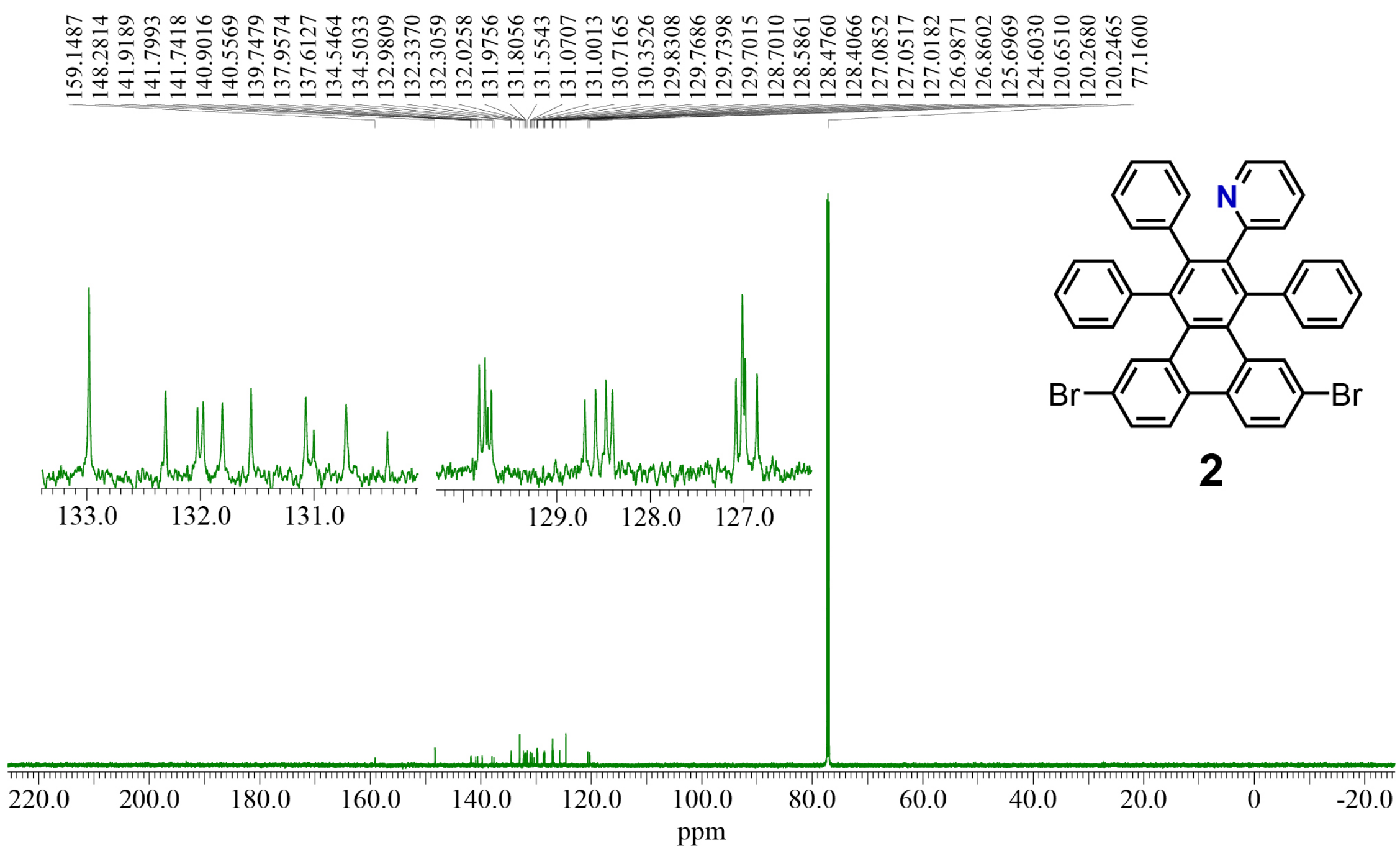

Figure S10: ^{13}C NMR spectrum of compound **2** ($CDCl_3$, 126 MHz, 298 K).

Scanning probe measurements and calculations

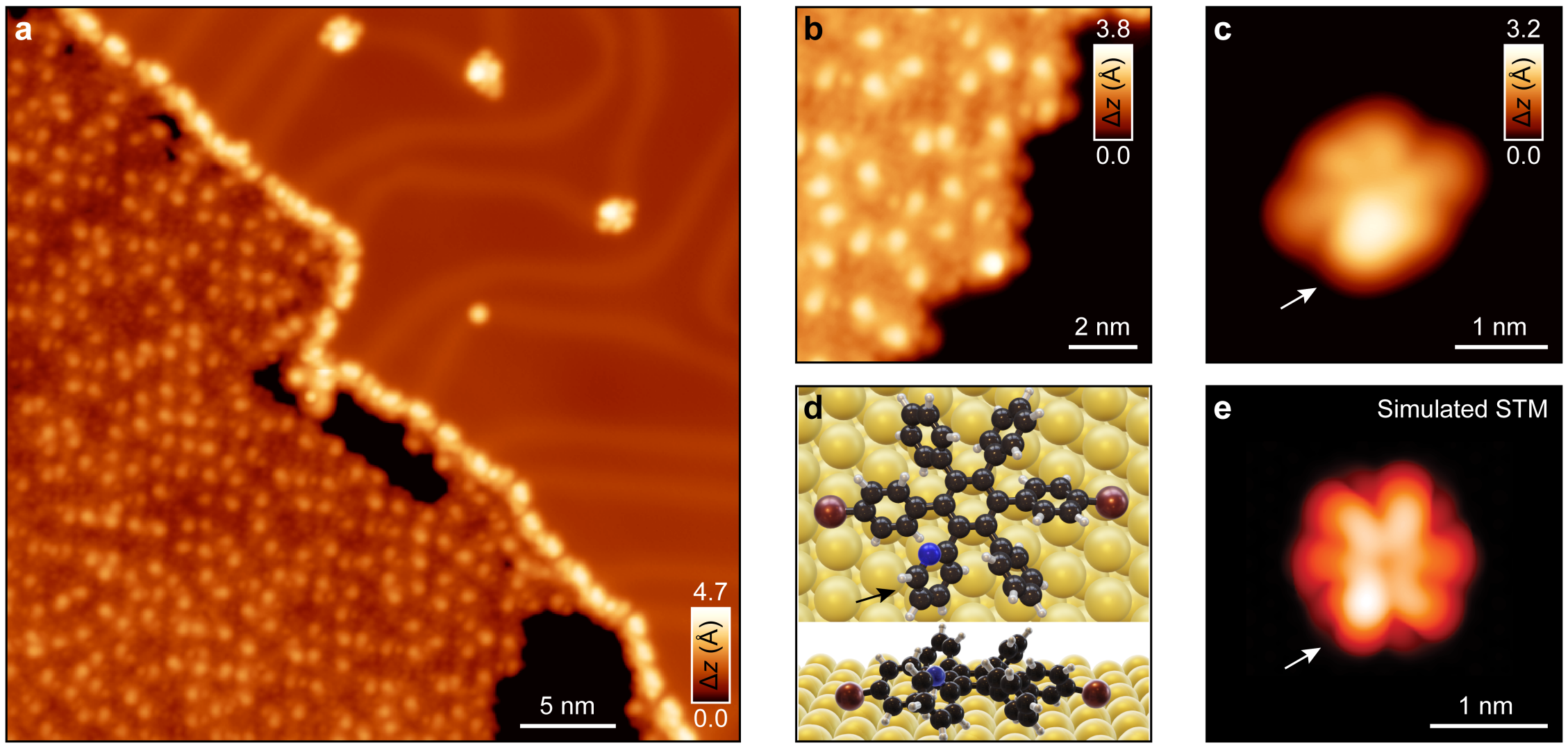

Figure S11: Characterization of **1** on Au(111). (a) STM overview image of the sample after deposition of **1** on Au(111) held at room temperature ($V = -1.5$ V, $I = 50$ pA). The surface consists of self-assembled molecular islands (lower terrace), and individual molecules anchored at the Au(111) herringbone elbow sites (upper terrace) and along the step edges. (b) High-resolution STM image of a self-assembled island ($V = -0.4$ V, $I = 100$ pA). (c) High-resolution STM image of **1** ($V = -1.0$ V, $I = 200$ pA). Six lobes corresponding to the outer non-planar rings of the molecule are visible in the STM image. The pyridyl ring, which appears brighter due to the higher density of states at the nitrogen site, is indicated by an arrow. (d) Top and side views of the adsorption geometry of **1** on Au(111) optimized using DFT. The pyridyl ring is indicated by an arrow. (e) Simulated STM image of **1** on Au(111) ($V = -1.1$ V, $I = 50$ pA). The enhanced contrast at the pyridyl ring (indicated by an arrow) is in good agreement with the experimental observation in (c).

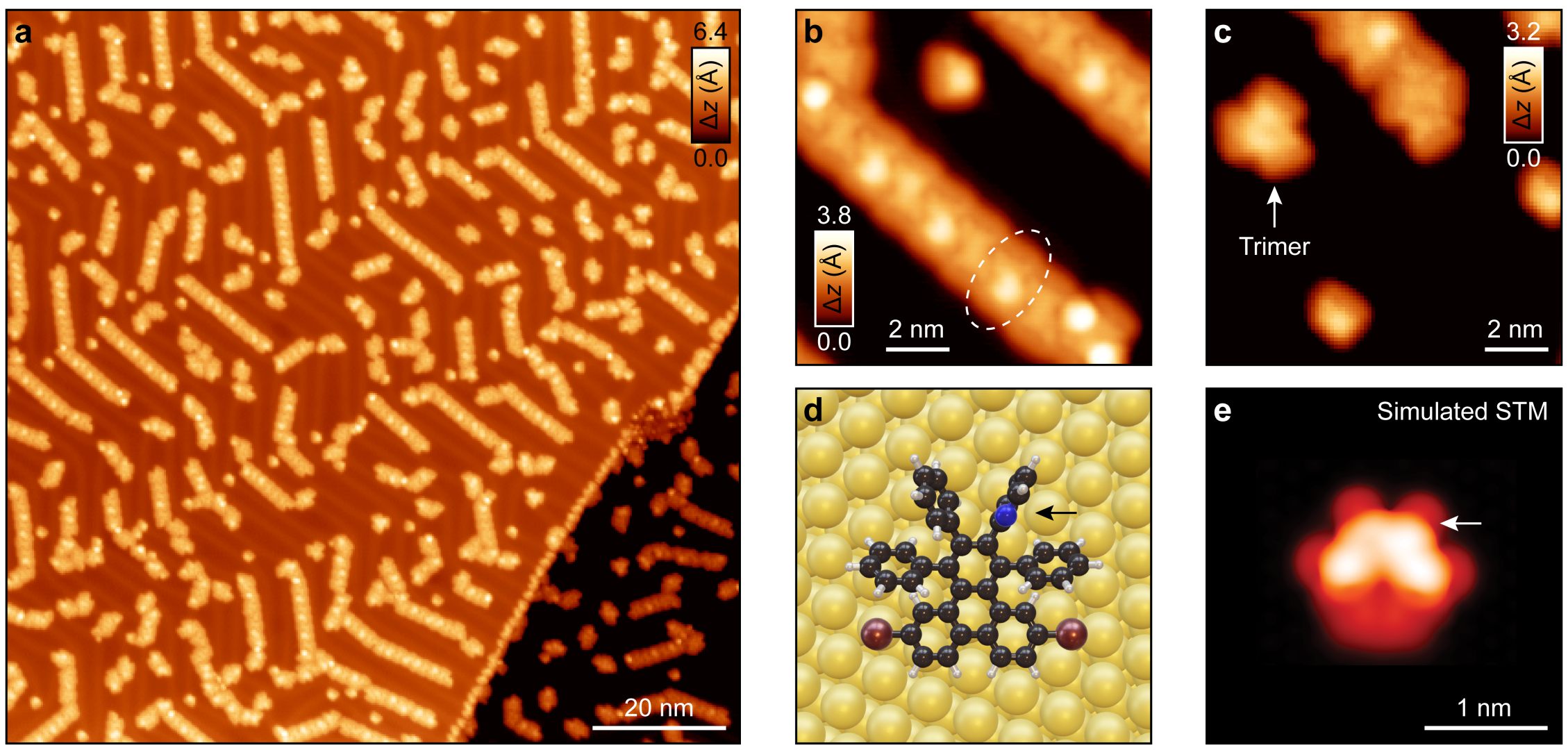

Figure S12: Characterization of **2** on Au(111). (a) STM overview image of the sample after deposition of **2** on Au(111) held at room temperature ($V = -1.5$ V, $I = 50$ pA). The surface consists of self-assembled chains on the fcc regions of the Au(111) surface, self-assembled clusters, and individual molecules anchored at the Au(111) herringbone elbow sites and along the step edges. (b) High-resolution STM image of self-assembled chains and an individual molecule ($V = -1.5$ V, $I = 50$ pA). The chains consist of repeating dimers of **2**, with a constituent dimer indicated by the dashed ellipse. Similar to **1**, the pyridyl ring of **2** appears brighter in STM imaging. (c) High-resolution STM image of molecular clusters and individual molecules ($V = -1.5$ V, $I = 50$ pA). A self-assembled trimer is indicated by an arrow. (d) Top view of the adsorption geometry of **2** on Au(111) optimized using DFT. (e) Simulated STM image of **2** on Au(111) ($V = -0.8$ V, $I = 100$ pA). The pyridyl ring is indicated by arrows in (d, e).

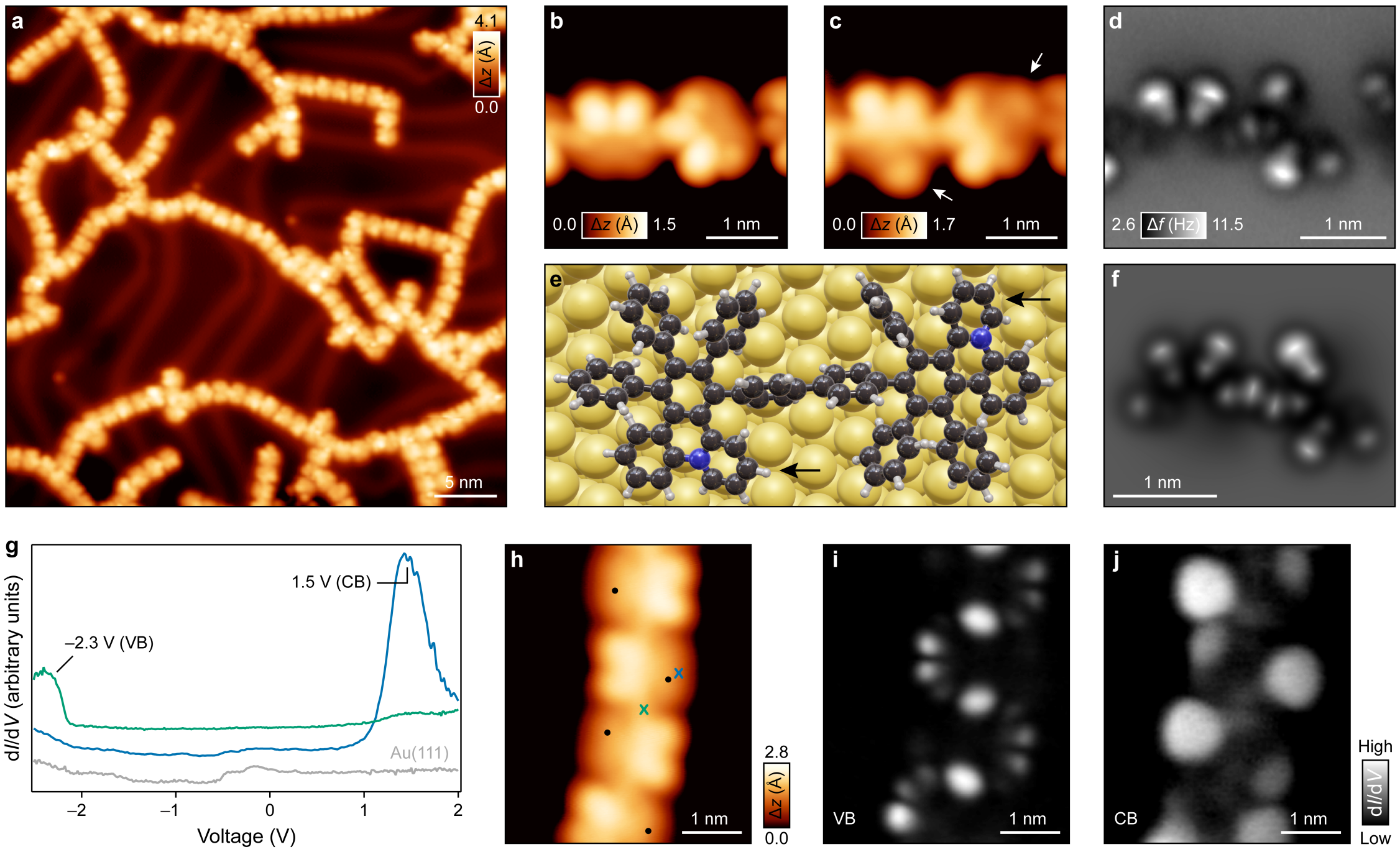

Figure S13: Characterization of *poly*-**1** on Au(111). (a) STM overview image after annealing **1** on Au(111) at 250 °C ($V = -1.2$ V, $I = 50$ pA). Ullmann-like coupling of **1** is evidenced by the presence of polymer chains on the surface. (b) High-resolution in-gap STM image of a *poly*-**1** segment ($V = -0.4$ V, $I = 15$ pA). Partial planarization of the individual units has occurred due to C–N bond formation. The non-planar phenyl rings appear as bright lobes. (c) High-resolution STM image of the same segment acquired at $V = 1.5$ V and $I = 15$ pA, revealing prominent lobes at the gN sites (indicated by arrows). (d) AFM image of the segment. The non-planar phenyl rings in each unit appear brighter (that is, with an increased Δf) due to stronger repulsive forces, whereas the comparatively planar pyrido[1,2-*a*]quinolin-11-ium moieties, which are adsorbed closer to the surface, are not resolved. The left and right units in (b–d) exhibit on- and off-axis C–N bond formation, respectively. STM set-point: $V = 5$ mV and $I = 100$ pA on Au(111); $\Delta z = 4.7$ Å. (e) Top view of the adsorption geometry of a dimer on Au(111) optimized using DFT and corresponding to the segment in (b–d). The nitrogen-containing six-membered rings are indicated by arrows. (f) Corresponding simulated AFM image of the dimer. (g) $\mathrm{d}I/\mathrm{d}V$ spectra acquired on a *poly*-**1** segment. Open feedback parameters: $V = -2.5$ V and $I = 300$ pA on the individual units; $V_{\mathrm{rms}} = 16$ mV. Acquisition positions are indicated by the colored crosses in the STM image in (h) ($V = -1.0$ V, $I = 130$ pA). Filled circles in (h) denote the positions of the nitrogen atoms. The spectra reveal two resonances at $V = -2.3$ V and $V = 1.5$ V, corresponding to the valence band (VB) and conduction band (CB), respectively. (i, j) Constant-current $\mathrm{d}I/\mathrm{d}V$ maps of the segment in (h) acquired at voltages corresponding to the resonances indicated in (g). Scanning parameters: $V = -2.3$ V, $I = 200$ pA (i), and $V = 1.5$ V, $I = 130$ pA (j); $V_{\mathrm{rms}} = 16$ mV. The state at $V = 1.5$ V is predominantly localized at the gN sites. The location of the state at positive bias indicates that it corresponds to an unoccupied nitrogen band. Thus, similar to *poly*-gN-HBC, *poly*-**1** adopts a cationic state on Au(111).

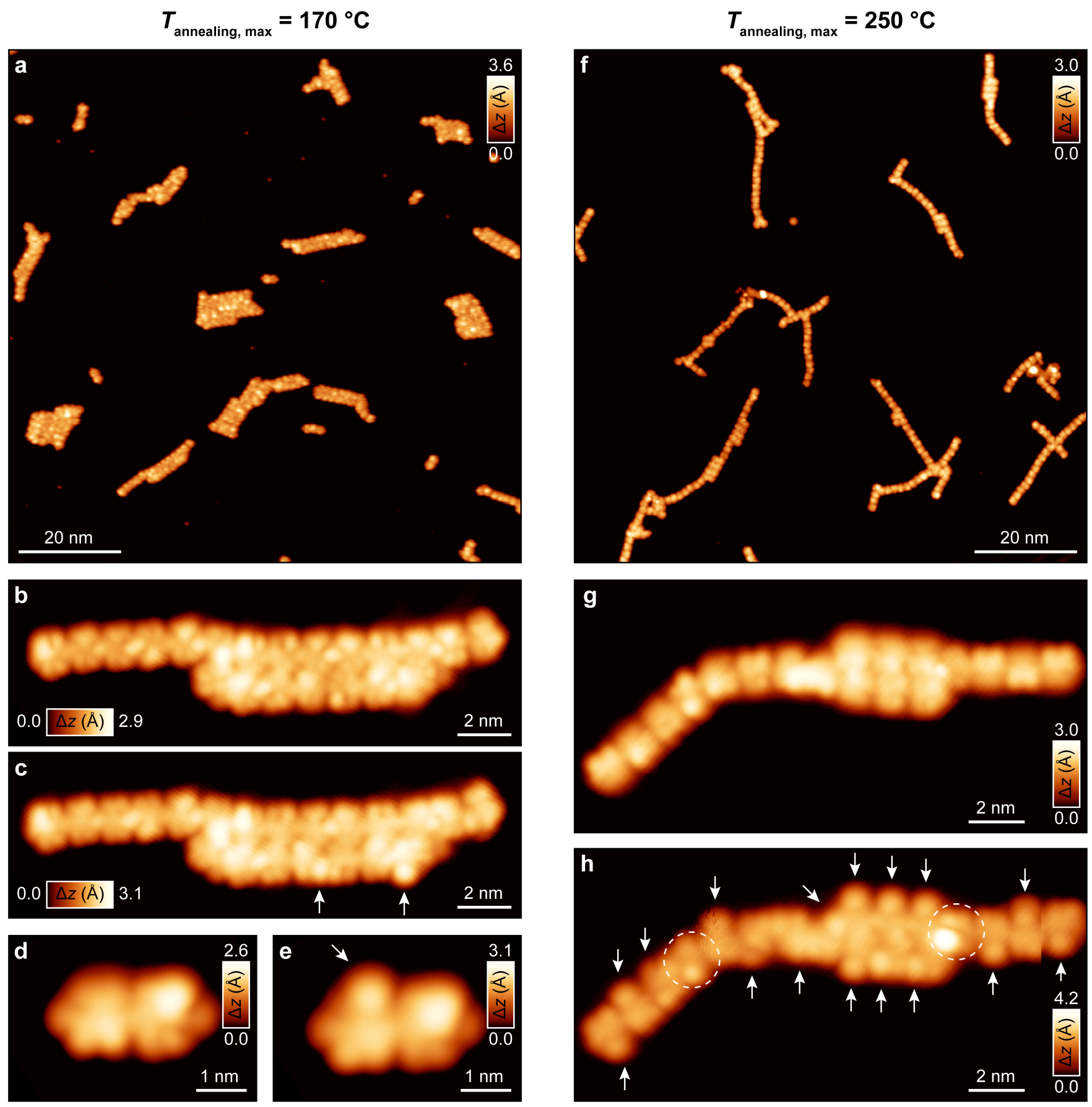

Figure S14: C–N bond formation as a function of annealing temperature. (a) STM overview image after annealing **1** on Au(111) at 170 °C for 20 minutes ($V = -1.0$ V, $I = 10$ pA). The surface mostly consists of self-assembled polymer chains and a few isolated short chains. (b) High-resolution in-gap STM image of two interdigitated polymer chains consisting of a total of 21 individual units ($V = -1.0$ V, $I = 60$ pA). (c) High-resolution STM image of the same structure acquired at $V = 1.5$ V and $I = 40$ pA. Only 2 out of the 21 units exhibit the characteristic lobes (indicated by arrows) associated with the gN sites, which is indicative of C–N bond formation. Analysis of multiple STM images revealed that 14 out of 136 units (∼10%) exhibited C–N bond formation at 170 °C. (d) High-resolution in-gap STM image of a dimer ($V = -0.2$ V, $I = 50$ pA), where the left unit exhibits planarization at the upper end, while the right unit shows the characteristic bright appearance of the pyridyl ring, as observed for **1** (Figure S11). (e) High-resolution STM image of the same dimer acquired at $V = 1.5$ V and $I = 40$ pA. The left unit exhibits the characteristic lobe associated with a gN site (indicated by an arrow). (f) STM overview image of the same sample after further annealing the sample at 250 °C for 15 minutes ($V = -1.0$ V, $I = 15$ pA). In contrast to (a), the polymer chains are no longer self-assembled but dispersed on the surface, indicating a high degree of planarization due to C–N bond formation. (g) High-resolution in-gap STM image of two interdigitated polymer chains consisting of a total of 18 individual units ($V = -1.0$ V, $I = 60$ pA). (h) High-resolution STM image of the same structure acquired at $V = 1.5$ V and $I = 2$ pA. Sixteen out of the 18 units exhibit the characteristic lobes associated with the gN sites (indicated by arrows), while only 2 units (indicated by dashed circles) do not exhibit these lobes. This indicates that C–N bond formation is nearly complete at 250 °C.

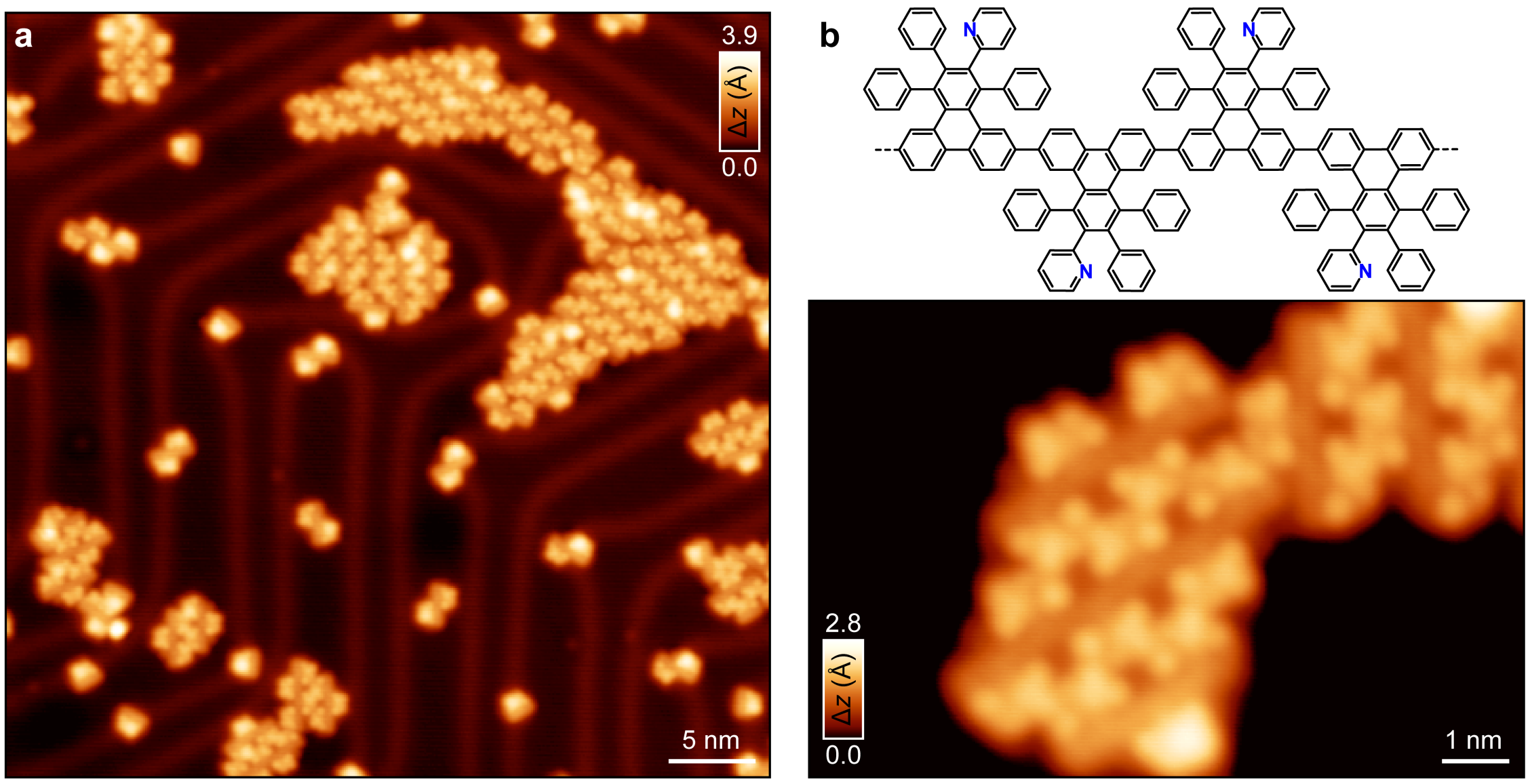

Figure S15: Characterization of an intermediate polymer phase after annealing **2** on Au(111). (a) STM overview image after annealing **2** on Au(111) at 180 °C ($V = -1.0$ V, $I = 50$ pA). The surface consists of self-assembled polymer chains, as well as isolated monomers to tetramers. The high degree of self-assembly indicates a general absence of C–N bond formation, consistent with the results shown in Figure S14 for a similar annealing temperature. (b) Chemical structure of the intermediate polymer (top) and high-resolution STM image of self-assembled polymer chains (bottom; $V = -0.7$ V, $I = 50$ pA). The central planar part of each polymer chain, flanked by features approximately 0.5 Å higher on either side, indicates that C–N bond formation has not occurred.

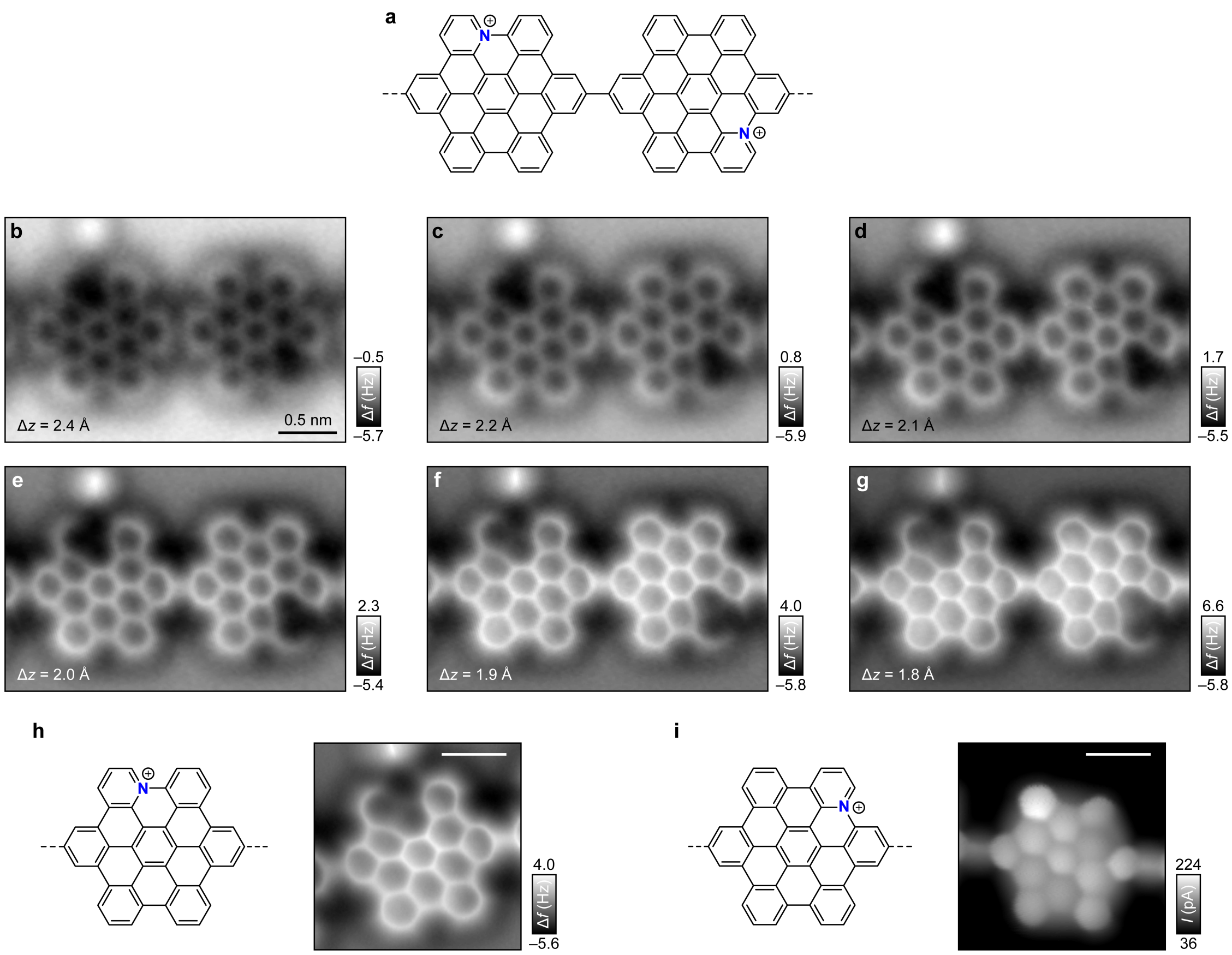

Figure S16: Bond-resolved STM and AFM imaging of *poly*-gN-HBC. (a) Chemical structure of a *poly*-gN-HBC segment consisting of two gN-HBC units. (b–g) High-resolution AFM images of the segment in (a) acquired at different Δz values, as indicated in the respective panels. STM set-point: $V = 5$ mV and $I = 100$ pA on Au(111). (h) Chemical structure (left) and high-resolution AFM image (right) of a gN-HBC unit. (i) Chemical structure (left) and high-resolution constant-height STM image acquired with a CO-functionalized tip (right) of a gN-HBC unit ($V = 5$ mV). Note the blurred appearance of the nitrogen-containing rings in (i). Scale bars: 0.5 nm.

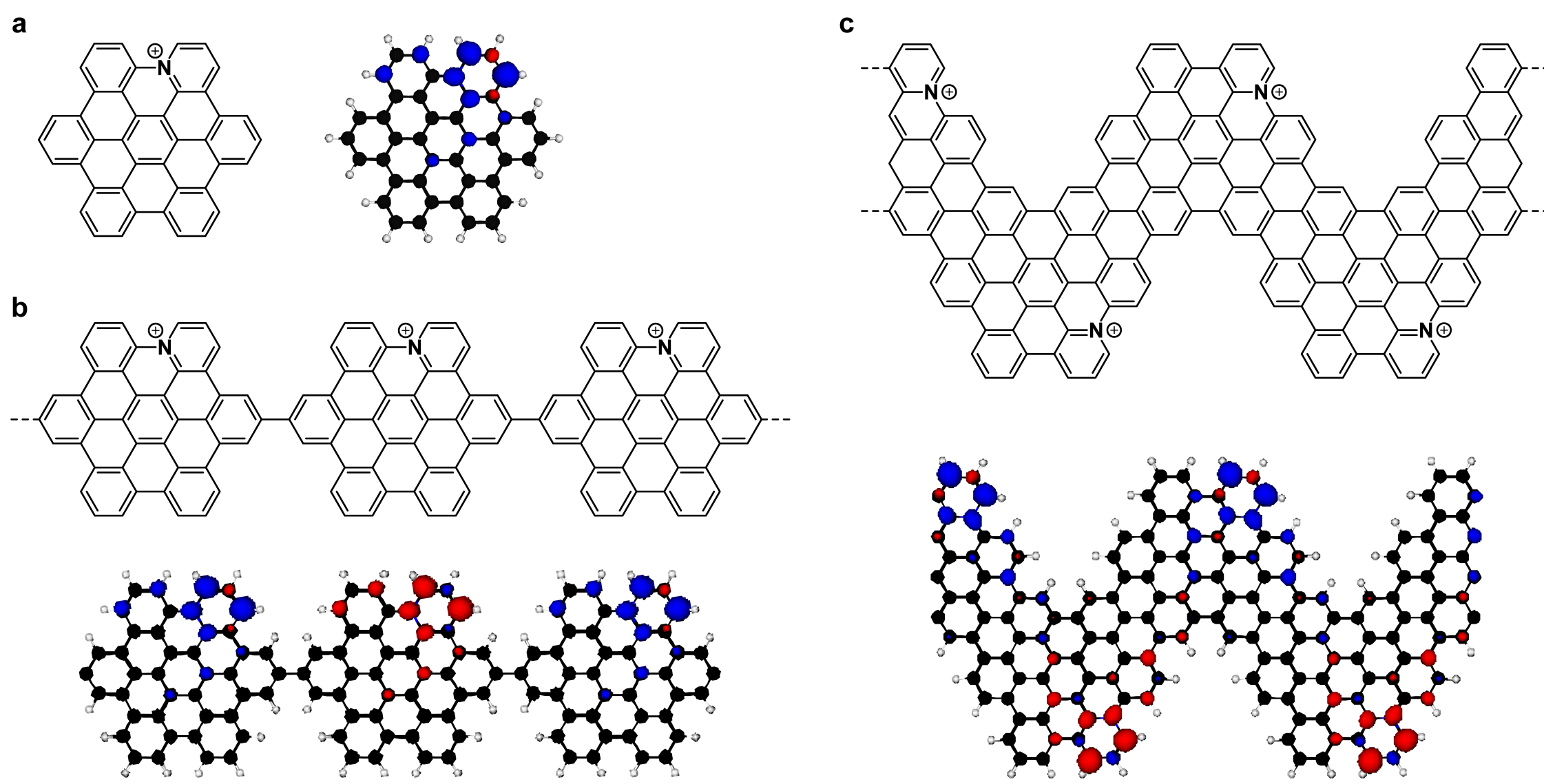

Figure S17: Spin-polarized DFT calculations. (a–c) Chemical structures and spin-density isosurface plots (obtained using DFT) of gN-HBC (a), *poly*-gN-HBC in the neutral antiferromagnetic state (b), and GNR-gN-HBC in the neutral antiferromagnetic state (c). The isovalue is 0.003 e/Bohr^3. Panel (a) is also shown in Figure 3c. The two colors in the spin-density isosurface plots represent spin up and spin down.

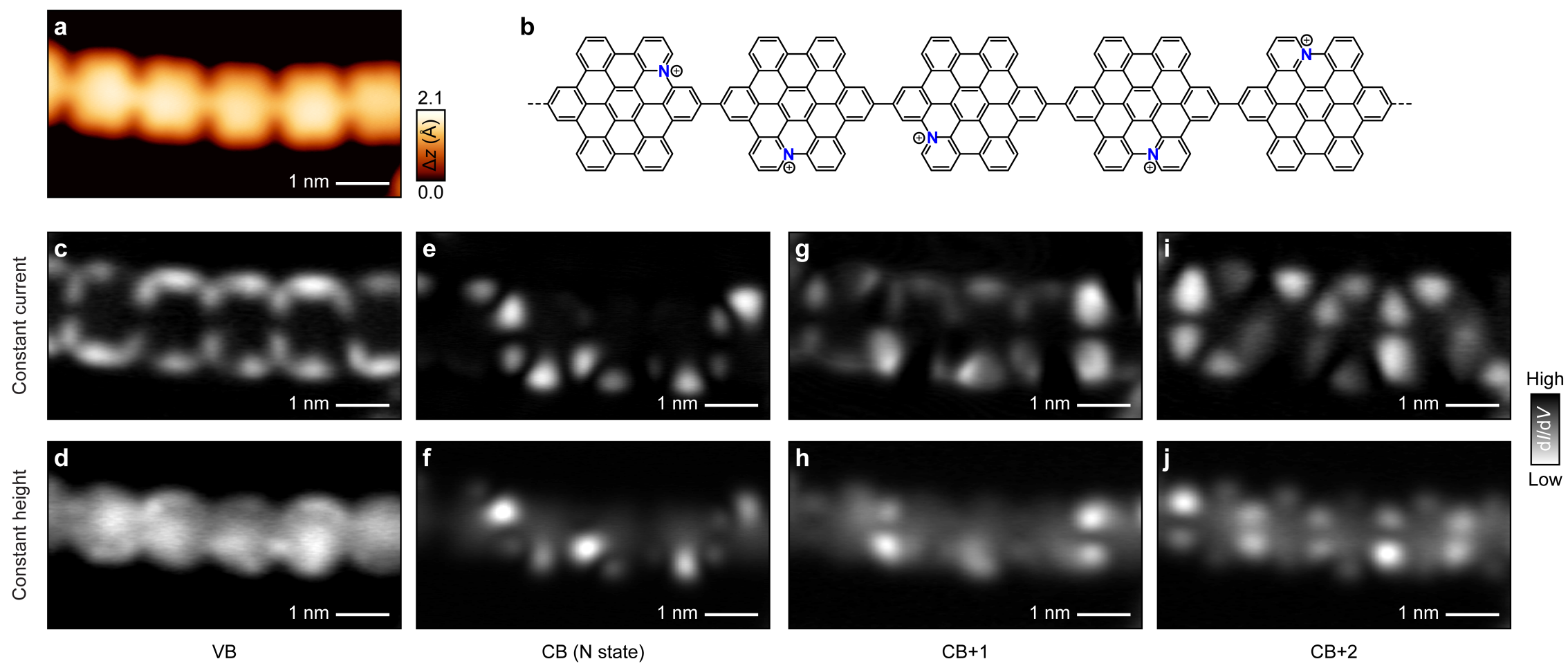

Figure S18: Constant-current and constant-height dI/dV maps of *poly*-gN-HBC. (a) High resolution STM image of a *poly*-gN-HBC segment ($V = -0.10$ V, $I = 200$ pA). (b) Chemical structure of the segment in (a). (c–j) Constant-current (top) and constant-height (bottom) dI/dV maps of the segment acquired at voltages corresponding to the resonances in Figure 4a: VB (c, d), CB (e, f), CB+1 (g, h) and CB+2 (i, j). Scanning parameters for the constant-current dI/dV maps: $V = -1.85$ V, $I = 250$ pA (c), $V = 0.70$ V, $I = 200$ pA (e), $V = 1.44$ V, $I = 200$ pA (g), and $V = 1.82$ V, $I = 200$ pA (i). STM set-point for the constant-height dI/dV maps: $V = -1.85$ V, $I = 300$ pA (d), $V = 0.90$ V, $I = 270$ pA (f), $V = 1.44$ V, $I = 250$ pA (h), and $V = 1.82$ V, $I = 280$ pA (j); $\Delta z = 0$ Å. The feedback loop was opened with the tip above the center of the third gN-HBC unit at the respective STM set-point currents and voltages, and the scanning voltages are the same as the set-point voltages. For all maps, $V_{\mathrm{rms}} = 16$ mV.

References

(1) Saleh, M.; Baumgarten, M.; Mavrinskiy, A.; Schäfer, T.; Müllen, K. Triphenylene-Based Polymers for Blue Polymeric Light Emitting Diodes. *Macromolecules* **2010**, *43*, 137–143, DOI: https://doi.org/10.1021/ma901912t.

(2) Gholinejad, M.; Neshat, A.; Zareh, F.; Najera, C.; Razeghi, M.; Khoshnood, A. Palladium Supported on Bis(indolyl)methane Functionalized Magnetite Nanoparticles as an Efficient Catalyst for Copper-Free Sonogashira-Hagihara Reaction. *Applied Catalysis A: General* **2016**, *525*, 31–40, DOI: https://doi.org/10.1016/j.apcata.2016.06.041.
